# Genomic Analysis of a *mcr-9.1*-Harbouring IncHI2-ST1 Plasmid from *Enterobacter ludwigii* Isolated in Fish Farming

**DOI:** 10.3390/antibiotics11091232

**Published:** 2022-09-10

**Authors:** Vera Manageiro, Vanessa Salgueiro, Tânia Rosado, Narcisa M. Bandarra, Eugénia Ferreira, Terry Smith, Elsa Dias, Manuela Caniça

**Affiliations:** 1 National Reference Laboratory of Antibiotic Resistances and Healthcare Associated Infections, Department of Infectious Diseases, National Institute of Health Dr. Ricardo Jorge, 1649-016 Lisbon, Portugal; 2 Centre for the Studies of Animal Science, Institute of Agrarian and Agri-Food Sciences and Technologies, University of Porto, 4051-401 Porto, Portugal; 3 AL4AnimalS, Associate Laboratory for Animal and Veterinary Sciences, 1300-477 Lisboa, Portugal; 4 Laboratory of Biology and Ecotoxicology, Department of Environmental Health, National Institute of Health Dr. Ricardo Jorge, 1649-016 Lisbon, Portugal; 5 Division of Aquaculture, Upgrading and Bioprospecting, Portuguese Institute for the Sea and Atmosphere, IPMA, 1749-077 Lisbon, Portugal; 6 CIIMAR, Interdisciplinary Centre of Marine and Environmental Research, University of Porto, Terminal de Cruzeiros do Porto, Av. General Norton de Matos s/n, 4450-208 Matosinhos, Portugal; 7 Molecular Diagnostics Research Group, School of Biological and Chemical Sciences, National University of Ireland, H91 DK59 Galway, Ireland; 8 Centre for One Health, Ryan Institute, National University of Ireland, H91 TK33 Galway, Ireland; 9 CIISA, Center for Interdisciplinary Research in Animal Health, Faculty of Veterinary Medicine, University of Lisbon, 1300-477 Lisbon, Portugal

**Keywords:** aquaculture, *mcr-9* gene, seabream, One Health

## Abstract

This study analyzed the resistome, virulome and mobilome of an MCR*-9*-producing *Enterobacter* sp. identified in a muscle sample of seabream (*Sparus aurata*), collected in a land tank from multitrophic fish farming production. Average Nucleotide Identity analysis identified INSAq77 at the species level as an *Enterobacter ludwigii* INSAq77 strain that was resistant to chloramphenicol, florfenicol and fosfomycin and was susceptible to all other antibiotics tested. In silico antimicrobial resistance analyses revealed genes conferring in silico resistance to β-lactams (*bla*_ACT-88_), chloramphenicol (*catA4-*type), fosfomycin (*fosA2-*type) and colistin (*mcr-9.1*), as well as several efflux pumps (e.g., *oqxAB-*type and *mar* operon). Further bioinformatics analysis revealed five plasmid replicon types, including the IncHI2/HI2A, which are linked to the worldwide dissemination of the *mcr-9* gene in different antibiotic resistance reservoirs. The conserved nickel/copper operon *rcnR-rcnA-pcoE*-IS*Sgsp1-pcoS-IS903-mcr-9-wbuC* was present, which may play a key role in copper tolerance under anaerobic growth and nickel homeostasis. These results highlight that antibiotic resistance in aquaculture are spreading through food, the environment and humans, which places this research in a One Health context. In fact, colistin is used as a last resort for the treatment of serious infections in clinical settings, thus *mcr* genes may represent a serious threat to human health.

## 1. Introduction

The emergence of colistin resistance in the last years is a serious threat to the treatment of infections caused by multidrug-resistant bacteria in human medicine. Consequently, colistin, a last resort antibiotic, is categorized by the World Health Organization (WHO) as one of the highest priority, critically important antibiotics for human medicine. The use of colistin in veterinary medicine has been prohibited in various countries. However, colistin is still an antibiotic extensively used in veterinary medicine for infections caused by *Enterobacterales* [1]. In aquatic animal species, colistin is also used to treat bacterial infections. Nevertheless, its application in aquaculture is much less common than that used in terrestrial animals [1].

Acquired resistance to colistin has been mostly due to chromosomal mutation in the PmrA/PmrB and PhoP/PhoQ, two-component regulatory systems, or through the increased production of capsular polysaccharide [2,3]. Since the first report of the plasmid-mediated colistin resistance (PMCR) gene *mcr-1* in an *Escherichia coli* strain isolated from a pig in China, we have seen an increase in the number of cases worldwide, in different bacterial species [4,5]. To date, ten PMCR genes (*mcr-1* to *mcr-10*), including variants, have been described, mainly in *Enterobacterales* from animals, humans and the environment [6]. Originally, MCR-producing bacteria were mostly isolated from animals, including pigs [7]. The exception are the *mcr-9* and *mcr-10* that were identified in human isolates: *Salmonella enterica* serotype Typhimurium and *Enterobacter roggenkampii*, respectively [8,9].

Unlike the situation in terrestrial animals, PMCR in aquaculture has largely been ignored [10]. However, colistin-resistant bacteria have also been found in aquaculture [11,12], being considered by the authors as a major source of colistin resistance genes. Recent studies in China showed the presence of MCR-1- and MCR-3-producing bacteria isolated in the food chain, such as in aquaculture fish and shrimp [13] and in *E. coli* recovered from grass carp fish farms [14].

In Vietnam, *mcr-1* was detected in *E. coli* isolated from striped catfish grown in ponds [15,16] and a study from Spain reported *mcr-1* in *S. enterica* serovar Rissen isolated from mussels [17]. Recently, *mcr-1* was detected in *E. coli* isolated from fish guts of rainbow trout in Lebanon [18]. In Czech Republic, *mcr*-type genes were detected in colistin-resistant *Enterobacterales* and *Acinetobacter* strains isolated from aquaculture products (frog legs, crab meat and pangasius meat) originating from Vietnam [19]. Shen and co-workers also demonstrated the association between aquaculture and a high incidence of *mcr-1*-positive *E. coli* carried by humans [13]. Furthermore, a comparative analysis of the resistome of integrated and monoculture aquaculture ponds using metagenomics suggest that freshwater aquaculture is rich in opportunistic pathogen-associated taxonomic groups that may host antibiotic-resistant genes (including *mcr*) associated with critically important antibiotics used in human medicine [20]. Indeed, it has been proposed that some PMCR genes may have originated in aquatic environments, since MCR-3, MCR-4 and MCR-7 proteins showed an elevated level of homology to phosphoethanolamine transferases found in aquatic bacteria [10,11].

The *mcr-9* gene is an emerging variant of the PMCR determinants, which was first identified in 2019, in a *S. enterica* isolated from a human patient in the USA [8]. Along with *mcr-1*, among the *mcr*-like genes, *mcr-9* is the most widely disseminated [7]. The *mcr-9* gene can be found worldwide in different reservoirs (human, animal, food and environment) and in various species of *Enterobacteriaceae* [21], which makes this resistance mechanism a problem under the perspective of One Health.

This study aimed to analyse the resistome, virulome and mobilome of an MCR*-9*-producing *Enterobacter* sp. isolated from farmed *Sparus aurata* and, to our knowledge, this is the first description of the colistin resistance *mcr-9* gene in the aquaculture environment.

## 2. Results and Discussion

The *mcr-1* and *mcr-9* variants are the most widespread *mcr*-family genes. The *mcr-9* gene was identified in 40 countries through six continents, with 61.5% of the *mcr-9*-positive strains isolated in the United States [7]. In that study, *S. enterica* was the most common host species, especially in turkeys and chickens. Furthermore, other systematic reviews showed that isolates carrying *mcr-9* were detected in 21 countries through six continents, mainly from Europe. *mcr*-9-positive isolates were disseminated by various genera and species of *Enterobacteriaceae* isolates among which *Enterobacter* spp. were predominant (37.0%) [21]. More than 50% of the isolates were from human origin, being 29.0%, 3.6% and 2.9% from animal, environmental and food, respectively. 

Here, an *mcr-9*-producing isolate (INSAq77) identified in a muscle sample of a commercial size *S. aurata*, collected during the winter season (March 2018), in a land tank from a fish multitrophic farming, in the south of Portugal is described. To our knowledge is the first description of *mcr-9* gene in the aquaculture environment. 

Average Nucleotide Identity (ANI) analysis performed by NCBI identified INSAq77 at the species level as *E. ludwigii*. The genome sequences of *E. cloacae* are 98.98% identical by ANI to the *E. ludwigii*, with 82.5% coverage of the genome. Indeed, INSAq77 isolate was identified as *E. cloacae* by the VITEK^®^2 automated identification system (BioMérieux, Marcy-l’Étoile, France) and sequencing of the 16S rRNA gene. However, it is well known that precise species identification for the taxonomy of *Enterobacter* is complex [22] and that *hsp*60 gene sequencing showed a higher species diversity than MALDI-TOF [23]. Seven species have been grouped within the *E. cloacae* complex: *Enterobacter cloacae*, *Enterobacter asburiae*, *Enterobacter hormaechei*, *Enterobacter kobei*, *E. ludwigii*, *Enterobacter mori* and *Enterobacter nimipressuralis*, which share at least 60% similarity in their genome with *E. cloacae* [24]. 

*E. ludwigii* was first described as a novel *Enterobacter* species in 2005 [25]. All strains are naturally resistant to ampicillin, amoxicillin-clavulanic acid, and cefoxitin due to the production of a chromosomal AmpC β-lactamase. Antibiotic-resistant *E. ludwigii* has been found mainly in clinical samples [26], although a CTX-M-producing *E. ludwigii* was recently described in an environmental isolate collected from a wastewater treatment plant in India [27]. Recently, antibiotic-resistant *E. ludwigii* was also identified in India, in moribund goldfish collected from ornamental fish farms [28]. On the other hand, *E. ludwigii* has been suggested as a potential probiotic microorganism in agriculture and aquaculture [29,30]. 

INSAq77 strain was resistant to chloramphenicol (MIC 32 mg/L), florfenicol (32 mg/L) and fosfomycin (64 mg/L) but was susceptible to all other antibiotics tested; cefoxitin and amoxicillin/clavulanic acid are intrinsic resistances. The colistin MIC for MCR*-9*-producing *E. ludwigii* as 1 mg/L, within susceptible EUCAST breakpoint. Indeed, other studies have demonstrated that the presence of an MCR-9 enzyme not always is associated with colistin resistance [8,31,32]. Nevertheless, recent studies showed that *mcr* genes might enhance the survival ability of bacteria under clinical colistin pressure, thereby potentially leading to treatment failure [33,34].

This study also analyzed the resistome, virulome and mobilome of this MCR-9-producing *E. ludwigii* isolated from farmed *Sparus aurata*. The analysis of WGS yielded 225 contigs, ranging from 237 to 244,787 bp. The draft genome contained a total assembly length of 5,276,953 bp, with estimated depth coverage of 30.7×; the GC content was 54.1%.

**Figure 1:**
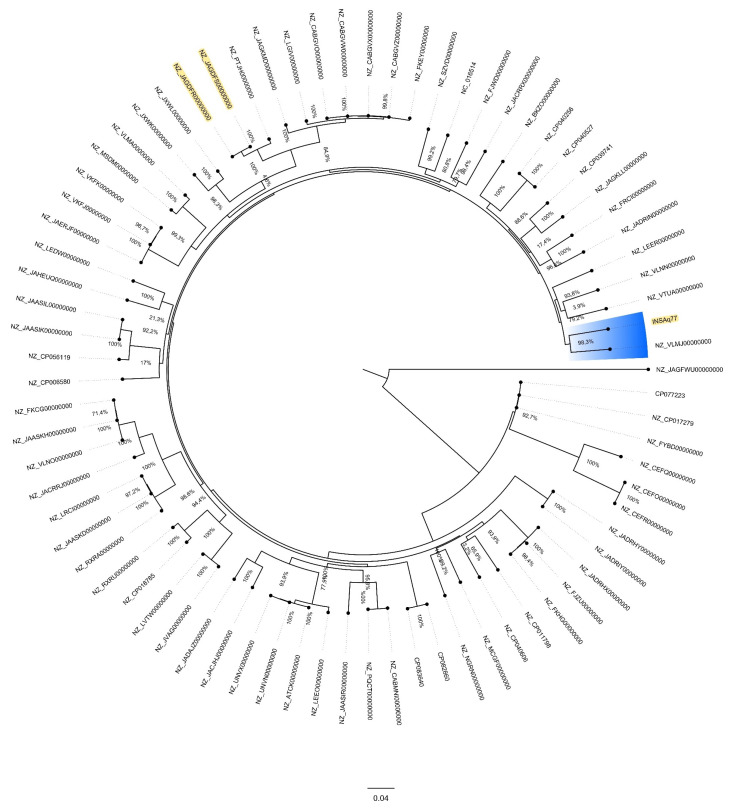
Whole-genome SNP-based phylogenetic tree showing the relationship between 75 *E. ludwigii* genomes. The scale bar indicates 4% of nucleotide sequence divergence. The numbers at the nodes indicate percentage bootstrap replicates of 100. Sequences in the tree are indicated as GenBank accession number. Strain of the present study and the other MCR-9-producing isolates are highlighted in yellow. Blue colour indicates cluster containing INSAq77.

The MCR-9-producing INSAq77 *E. ludwigii* isolate belonged to the ST1342 lineage, first reported here. The whole-genome SNP-based phylogenetic tree using the 75 *E. ludwigii* genomes indicated that INSAq77 is not closely related to the other studied isolates (Figure 1).

Indeed, INSAq77 has 12% of nucleotide sequence divergence with the closest strain (NZ_VLMJ00000000), an *E. ludwigii* isolated from the lung of a clinical patient, in 2016, in the USA (PRJNA553678) [35]. The two other MCR-9-producing isolates (NZ_JAGDFR00000000 and NZ_JAGDFs00000000) were grouped into another cluster.

In silico antimicrobial resistance analyses using ResFinder 4.1, with a threshold of 90% identity and a minimum length of 60%, revealed acquired genes conferring resistance to β-lactams (*bla*_ACT-88_, here firstly identified), fosfomycin (*fosA2*-type) and colistin (*mcr-9.1*). Furthermore, a total of 21 genes were detected in silico by CARD RGI perfect, strict and loose algorithms, involved in efflux, transport and permeability, which might justify the florfenicol and chloramphenicol resistance identified by phenotypic methods (Table 1 and Table S1). 

CARD *loose* algorithm (match bitscore less than the curated one blastp bitscore) [36] identified that INSAq77 also harbors a *catA4*-type gene (64.8% of identity), which might infer resistance to chloramphenicol. However, the low percentage of identity it is not enough to assure the phenicol resistance causality of *catA4*-type gene. Other resistance mechanisms might be involved; indeed, the multiple antibiotic resistance (*mar*) locus, a resistance-nodulation-cell division (RND) antibiotic efflux pump detected in silico by CARD RGI strict algorithm, has been reported to contribute to chloramphenicol resistance [37]. Furthermore, the multiple antibiotic resistance *oqxAB*-type locus, another RND multidrug efflux pump operon was detected, which has been reported to contribute to multidrug resistance [38]. Diminished susceptibility to different antibiotic classes (e.g., aminoglycosides, fluoroquinolones and tetracycline) were bioinformatically predicted (e.g., *rsmA*, *adeF, ramA, acrABR* and *soxAS*), although the isolate was phenotypically susceptible; this can be explained by the fact that efflux pumps are frequently associated with a low decrease in antibiotic susceptibility, which may not translate to a change in phenotype [39]. 

The acquired disinfectant resistance gene *formA*-type, a plasmid-mediated formaldehyde resistance mechanism [40], was also identified. The ability to survive aldehyde disinfection processes is clinically significant, with possible cross-resistance to antibiotics [41]. Furthermore, the INSAq77 isolate carried the *terC* virulence gene, commonly associated with IncHI2 plasmids and conferring resistance to tellurium, where soluble salts, especially potassium tellurite, were used clinically in humans as antimicrobial agents [42]. 

PathogenFinder predicted the strain as being “human pathogenic” with a probability of 77.7% due to the presence of 74 genes belonging to known pathogenic protein families (Table S2). Indeed, in addition to the known *E. cloacae* complex genes encoding pathogenic proteins and the homologous sequences of pathogenic proteins from *Citrobacter koseri*, *Enterobacter* spp., *E. coli*, *S. enterica* and *Shigella* spp. were found in the study. Mobile genetic elements (MGE), such as plasmids, prophages and transposons among others, are main drivers for the spread of antibiotic resistance [43]. In this study, nine insertion sequences (IS) were found using MobileElementFinder tool: IS*26*, IS*Kpn28*, IS*Sen4*-type, IS*30*-type, IS*Ecl1*-type, IS*Kpn43*-type, IS*Kpn24*-type, IS*100-*type and IS*Ppu12*-type. 

**Figure 2:**
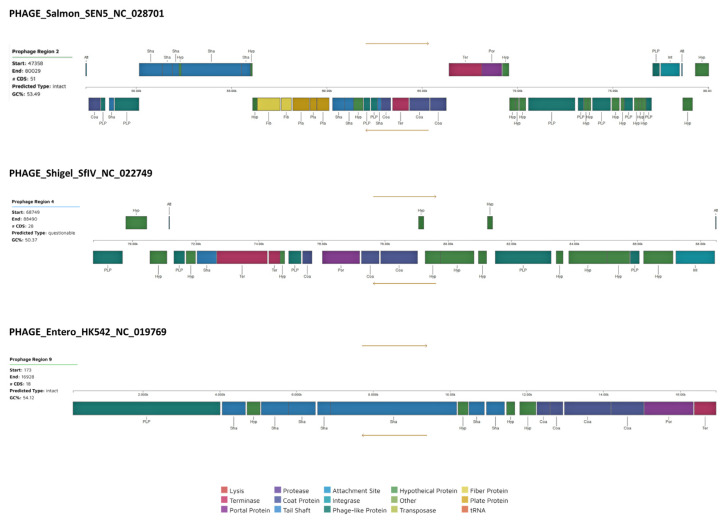
Schematic representation of phage-related proteins identified in the intact and questionable prophages by PHASTER prophage database (https://phaster.ca, accessed on 4 January 2022) [49]. The arrow indicates the sequence orientation (5′ to 3′ above the black line and 3′ to 5′ under it). The abbreviations are: Att (phage attachment site), Coa (Phage coat protein), Fib (Phage Tail Fibre), Int (Phage integrase), Hyp (Hypothetical protein), Pla (Phage plate protein), PLP (Phage-like protein), Por (Portal protein), Sha (Phage tail shaft protein) and Ter (Terminase).

A total of eleven prophage regions were also identified using PHASTER tool (Figure S1), of which two regions were intact (PHAGE_Salmon_SEN4_NC_029015 and PHAGE_Entero_HK542_NC_019769), eight regions were incomplete and one region was questionable (PHAGE_Shigel_SfIV_NC_022749). Figure 2 shows the schematic representation of the phage-related proteins identified in the intact and questionable prophages. The size of the three prophages ranged from 16.7Kb to 32.6Kp with an average GC content of 52.7%. These prophages were firstly described in *S. enterica* subspecies *salamae* collected in the Czech Republic [44], *E. coli* isolated in Hong Kong and *Shigella flexneri* collected in Bangladesh [45], corroborating that MGE can be excised and integrated from the chromosome and MGE into each other. Indeed, several studies have already shown the presence of *mcr-*type genes in prophages [46,47], indicating the role of these MGE in the dissemination of antibiotic resistance. Furthermore, two cryptic prophages were detected by PathogenFinder (CP4-6 and CP4-57) which, although they do not form active phage particles or lyse their captors, can be considered the relatively permanent reservoirs of antibiotic resistance genes [48].

Further bioinformatics analysis revealed the presence of five plasmid replicon types: ColE10, Col(pHAD28)-type, IncFIA(HI1)-type, IncR-type, IncHI2 and IncHI2A, the last two linked to the worldwide dissemination of *mcr-9* gene [7,21]. The *mcr-9* gene can be found in different reservoirs (human, animal, food and environment), in various species of *Enterobacteriaceae* strains, mostly associated with IncHI2/IncHI2A plasmid replicons [21]. Indeed, as observed in our study, several works demonstrated the prevalence of *mcr-9*-harboring IncHI2/IncHI2A plasmids among *Enterobacteriaceae* isolates: e.g., Carroll et al., in 2019, detected 59/65 assemblies where IncHI2 and/or IncHI2A plasmid replicon were present on the same contig as *mcr*-9 [8]. 

The *mcr-9* gene was found in a 30,314 bp length contig, manually assembled after visual inspection and alignment of contigs (Aq77p_57, Aq77p_155, Aq77p_191, Aq77p_196, Aq77p_213) against themselves using CLC Genomics Finishing Module v.20.0.1 (QIAGEN, Aarhus); the GC content was 47.6%. The analysis of *mcr-9*-harbouring contig using the Microbial Nucleotide MegaBLAST analysis against the complete plasmids database revealed nine *mcr-9*-carrying IncHI2 plasmid sequences (>99.9% identity, >97% query coverage and e-value 0.0) from multiple species, collected in different antibiotic resistance reservoirs worldwide, including human clinical/colonization samples (Table 2). Of notice, three plasmids of sequence type 1 (ST1), accordingly with the IncHI2 pDLST scheme, were collected from environmental samples during an extended *bla*_IMP-4_-associated carbapenemase outbreak in an Australian hospital [50].

**Figure 3:**
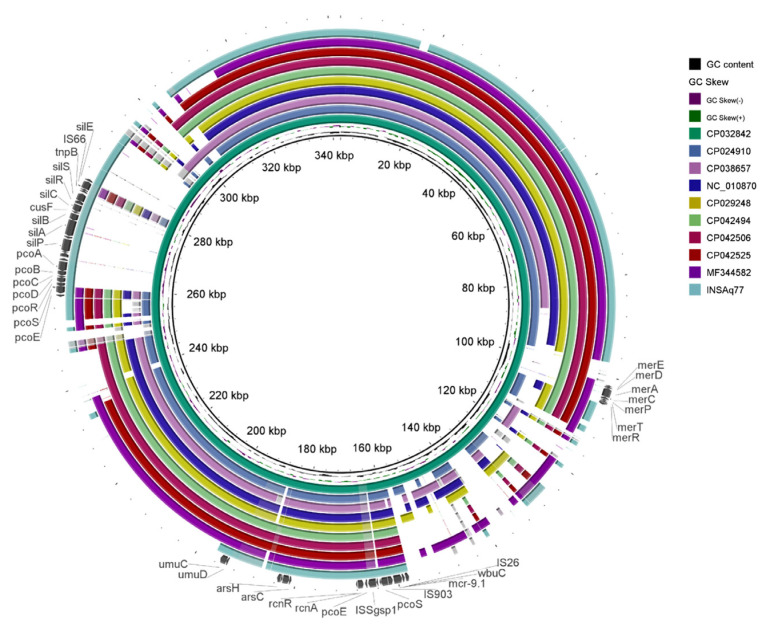
Comparative analysis of INSAq77 *mcr-9.1*-containing contig with nine closely related IncHI2 *mcr-9*-harboring plasmids using the BLAST Ring Image Generator.

In INSAq77 IncHI2 plasmid and in all others studied here, except p565_1 from a *C. freundii* strain (NZ_CP038657, Figure 3), the *mcr-9* gene was surrounded upstream by an IS*903* element and downstream by a *wbuC* family gene, encoding a cupin fold metalloprotein, followed by IS*26*. 

The *mcr*-9 gene is frequently associated with the *wbuC* gene in different bacterial species, suggesting an essential role of this *wbuC* gene for the activity of the MCR-9 enzyme [51]. Indeed, this gene was proposed to have transferred together with *mcr-9* as a whole fragment from *Buttiauxella* spp. [51]. On the other hand, *qseB/qseC* two-component regulators were absent, which could explain the susceptibility to colistin of INSAq77 isolate. Indeed, based on previous studies, in the presence of subinhibitory concentrations of colistin, the *qseB/qseC* regulatory system can induce the expression of the *mcr-9* gene, which results in an increase in MIC values [32,51,52]. The *mcr*-9 gene is being described as part of a *mcr-9* cassette containing the *rcnR-rcnA-pcoE-pcoS-IS*903*-mcr-9*-*wbuC* core structure (Figure 3) [21].

**Figure 4:**
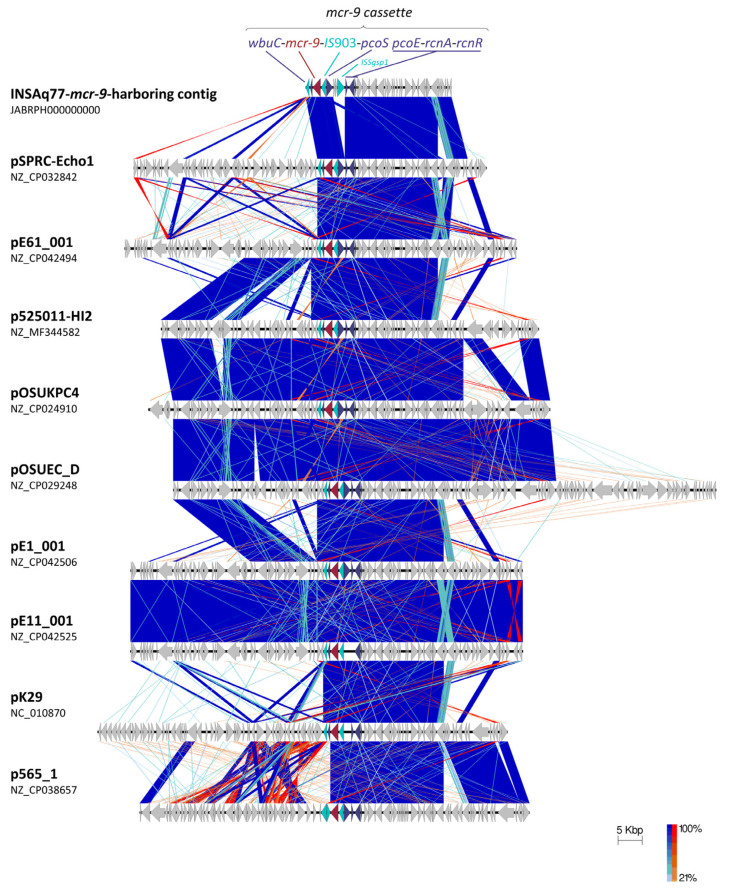
Schematic representation of the genetic environment of INSAq77 *mcr-9.1*-containing contig with others IncHI2 *mcr-9*-harboring plasmids. Boxed arrows indicate direction of transcription for all genes. Blue bars: normal tblastx matches; red: Inverted matches; depth of shading: percentage blast match. Color-coding for the genes inside *mcr-9* cassette: dark red, *mcr-9* gene; cyan, mobile DNA; purple, other genes; grey, other CDSs. The scale is represented in base pairs.

As shown in Figure 3 and Figure 4, the genetic background immediately upstream of *mcr-9* was consistent among IncHI2 *mcr-9*-bearing plasmids. The exception is the presence of an IS*Sgsp1* element from the IS*66* family in the INSAq77 *mcr-9*-harbouring contig, showing 100% of identity with an isolate of *Klebsiella pneumoniae* collected, in 2012, from a human patient in the USA (CP007734). Furthermore, in this study, the presence of the conserved nickel/copper operon (i.e., *rcnA*, *rcnR*, *pcoE* and *pcoS* genes), which plays a key role in copper tolerance under anaerobic growth and nickel homeostasis in bacteria, was also detected. Downstream of the *mcr-9* gene, the nucleotide sequences, including that of INSAq77, were genetically diverse (Figure 3). Indeed, different regions were present among the IncHI2 plasmids also analyzed in this study, namely regions involved in resistance to copper (*pcoABCDRSE*), silver (*silESRCBAP*), arsenic (ars*CBRH*) and/or mercury (*merEDACPTR*).

The abundance of *mcr* variants and alleles in bacteria isolated from aquatic reservoirs suggests that these enzymes may play another role, namely a defense system against natural peptides and/or bacteriophages [53]. *mcr-9* gene was firstly described in the USA, in a clinical *S.* Typhimurium isolate, which demonstrates the high transmission potential of this colistin resistance determinant and places this research in a One Health context [54]. 

## 3. Materials and Methods

### 3.1. Study Design and Bacterial Identification

MCR-9-producing *Enterobacter* sp. INSAq77 was isolated from a seabream (*S. aurata*) of commercial-size (500–1500 g), which was collected in March 2018 in a land tank from a fish multitrophic farming [55]. This station is in the Ria Formosa Natural Park (south of Portugal) with a semi-intensive production system. Animal welfare was safeguarded during production and transport accordingly with the European Commission SANTE/2016/G2/009 recommendations [56]. Species identification was performed by VITEK^®^2 Automated Identification System (BioMérieux, Marcy-l’Étoile, France), using GN ID card and by amplification of the 16S rRNA gene, as previously described [55].

### 3.2. Antimicrobial Susceptibility Testing

Antibiotic susceptibility testing was performed by disk diffusion (amoxicillin/clavulanic acid, aztreonam, cefepime, cefotaxime, cefoxitin, ceftazidime, ciprofloxacin, ertapenem, gentamicin, imipenem, meropenem, piperacillin/tazobactam and trimethoprim/sulfamethoxazole; Bio-Rad, Marnes-la-Coquette, France) and minimum inhibitory concentration (MIC) by in house broth microdilution (colistin, chloramphenicol, florfenicol, flumequine and oxytetracycline) and E-test^®^ (fosfomycin; bioMérieux, Hazelwood, MO, USA), as previously described [55].

### 3.3. Whole-Genome Sequencing

DNA was extracted from freshly grown overnight culture (MagnaPure 96 Instrument, Roche, Manheim, Germany) and was quantified using Qubit fluorometer (Thermo Fisher Scientific, Waltham, MA, USA). Dual-indexed Nextera XT kit was used to library preparation followed by paired-end sequencing (2 × 250 bp) on a MiSeq Illumina platform (Illumina Inc., San Diego, CA, USA), according to the manufacturer’s instructions.

### 3.4. Genome Annotation and Analysis

Genomes were de novo assembled using the INNUca v4.2.2 pipeline (https://github.com/B-UMMI/INNUca; accessed on 4 January 2022): after quality control analysis performed by FastQC v0.11.5 and cleaning (Trimmomatic v0.38), genomes were assembled with SPAdes 3.14.0 and subsequently improved using Pilon v1.23. In silico multilocus sequence type (MLST) prediction was performed using the MLST v2.19.0. A Prokka v1.13.3 was utilized to annotate the assemblies. Average Nucleotide identity (ANI) was performed at NCBI to confirm the INSAq77 bacterial species [57]. All de novo contigs were BLAST searched against GenBank’s non-redundant nucleotide collection (nr/nt) [58]. QIAGEN CLC Genome Finishing Module v.20.0.1 (QIAGEN, Aarhus, Denmark) was used for visual inspection and manual editing by the alignment of contigs using BLAST against the contigs themselves, allowing contig joining and scaffolding.

### 3.5. Phylogenomic Analyses of E. ludwigii Genomes

All *E. ludwigii* genomes (*n* = 76, Table S3) available at NCBI library were used on the genomic comparison process. Single nucleotide polymorphisms (SNPs) phylogenetic analysis was performed by using CSI Phylogeny v1.4 (https://cge.cbs.dtu.dk/services/CSIPhylogeny/; accessed on 4 January 2022) with default options (reference strain NZ_CP017279). Phylogenetic tree image was visualized and edited by FigTree v1.4.4 (https://tree.bio.ed.ac.uk/software/figtree/; accessed on 4 January 2022).

### 3.6. Resistome, Virulome and Mobilome Analysis

Online bioinformatics tools and databases available at the Center for Genomic Epidemiology (CGE) (www.genomicepidemiology.org; accessed on 4 January 2022) were used to investigate the presence of antimicrobial resistance genes (ResFinder 4.1), virulence factors (VirulenceFinder 2.0), plasmids (PlasmidFinder 2.1 and pMLST 2.0 IncHI2 DLST configuration), mobile genetic elements (MobileElementFinder v1.0.3) and pathogenicity (PathogenFinder 1.1). The Comprehensive Antibiotic Resistance Database (CARD) with the “perfect”, “strict” and “loose” default settings were also used to characterize antibiotic resistance [36]. ISsaga was used for the identification and annotation of insertion sequences [59]. PHASTER search web tool (https://phaster.ca; accessed on 4 January 2022) was applied to detect, identify and annotate prophage sequences [49]. All analyses were performed using default parameters.

### 3.7. Plasmid Characterization

BRIG v.0.95 was used to perform a circular comparison between the complete sequence of INSAq77 *mcr-9*-harbouring contig and the highly similar plasmids detected by performing BLAST against the Microbial Nucleotide BLAST database for complete plasmids (https://blast.ncbi.nlm.nih.gov/Blast.cgi; accessed on 4 January 2022). The genetic environment of the *mcr-9* gene was manually revisited using CLC Genomics Workbench v20.0.4 (QIAGEN, Aarhus, Denmark). EasyFig v2.2.5 was used for the visualization and comparison of *mcr-9* genetic environment [60].

## 4. Conclusions

This work reinforces the knowledge that water environments play a crucial role in the spread of antibiotic resistance and that important antibiotic resistance mechanisms, such as *mcr* genes conferring low- or medium/high-level resistance to colistin, are also present in aquaculture. This fact, allows antibiotic-resistant bacteria to spread through food and through the environment, resulting in serious threats to human health [61]. 

The use of phenotypic methods to determine susceptibility to antibiotics may be a limitation, as they may not identify the low expression associated with the presence of a particular gene, as in this case. Thus, the implementation of high throughput methods in laboratories, such as the WGS, will make an important contribution to the detection of under-expressed genes, mostly when they are of clinical importance. Thus, the presence of antibiotic-susceptible isolates in different settings, such as the INSAq77 *mcr-9*-carrying strain isolated in aquaculture, highlights the risk of the silent dissemination of important resistance determinants, among which, in fact, the genes encoding such PMCR are an important example. Of concern is also the possible co-selection of antibiotic-resistant bacteria when exposed to heavy metals (copper and zinc), often used as growth promoters in aquaculture and terrestrial animal farms.

## Figures and Tables

**Table 1:** Perfect and strict best-hit results by predicted gene, obtained using the Resistance Gene Identifier (RGI).

Contig	RGI Criteria	ARO Term	Detection Criteria Model	AMR Gene Family	Drug Class	Resistance Mechanism	% Identity Matching Region	% Length Reference Sequence
INSAq77p_155	Perfect	*mcr-9.1*	protein homolog	MCR phosphoethanolamine transferase	peptide antibiotic	antibiotic target alteration	100.0	100.0
INSAq77p_4	Strict	CRP	protein homolog	resistance-nodulation-cell division (RND) antibiotic efflux pump	macrolide antibiotic, fluoroquinolone antibiotic, penam	antibiotic efflux	99.1	100.0
INSAq77p_10	Strict	ACT-12	protein homolog	ACT beta-lactamase	carbapenem, cephalosporin, cephamycin, penam	antibiotic inactivation	98.7	100.0
INSAq77p_1	Strict	FosA2	protein homolog	fosfomycin thiol transferase	fosfomycin	antibiotic inactivation	98.6	100.0
INSAq77p_4	Strict	*Escherichia coli* EF-Tu mutants (R234F)	protein variant	elfamycin-resistant EF-Tu	elfamycin antibiotic	antibiotic target alteration	98.5	96.3
INSAq77p_82	Strict	baeR	protein homolog	resistance-nodulation-cell division (RND) antibiotic efflux pump	aminoglycoside antibiotic, aminocoumarin antibiotic	antibiotic efflux	95.8	100.0
INSAq77p_37	Strict	H-NS	protein homolog	major facilitator superfamily (MFS) antibiotic efflux pump, resistance-nodulation-cell division (RND) antibiotic efflux pump	macrolide antibiotic, fluoroquinolone antibiotic, cephalosporin, cephamycin, penam, tetracycline antibiotic	antibiotic efflux	95.6	100.0
INSAq77p_47	Strict	msbA	protein homolog	ATP-binding cassette (ABC) antibiotic efflux pump	nitroimidazole antibiotic	antibiotic efflux	94.7	100.0
INSAq77p_3	Strict	emrR	protein homolog	major facilitator superfamily (MFS) antibiotic efflux pump	fluoroquinolone antibiotic	antibiotic efflux	94.3	100.0
INSAq77p_25	Strict	*Escherichia coli* UhpT mutant (E350Q)	protein variant	antibiotic-resistant UhpT	fosfomycin	antibiotic target alteration	93.7	100.0
INSAq77p_21	Strict	marA	protein homolog	resistance-nodulation-cell division (RND) antibiotic efflux pump, General Bacterial Porin with reduced permeability to beta-lactams	fluoroquinolone antibiotic, monobactam, carbapenem, cephalosporin, glycylcycline, cephamycin, penam, tetracycline antibiotic, rifamycin antibiotic, phenicol antibiotic, triclosan, penem	antibiotic efflux, reduced permeability to antibiotic	93.6	99.2
INSAq77p_3_7	Strict	*Klebsiella pneumoniae* KpnH	protein homolog	major facilitator superfamily (MFS) antibiotic efflux pump	macrolide antibiotic, fluoroquinolone antibiotic, aminoglycoside antibiotic, carbapenem, cephalosporin, penam, peptide antibiotic, penem	antibiotic efflux	92.2	100.6
INSAq77p_11	Strict	oqxA	protein homolog	resistance-nodulation-cell division (RND) antibiotic efflux pump	fluoroquinolone antibiotic, glycylcycline, tetracycline antibiotic, diaminopyrimidine antibiotic, nitrofuran antibiotic	antibiotic efflux	91.1	100.0
INSAq77p_21	Strict	*Escherichia coli marR* mutant conferring antibiotic resistance	protein overexpression	resistance-nodulation-cell division (RND) antibiotic efflux pump	fluoroquinolone antibiotic, cephalosporin, glycylcycline, penam, tetracycline antibiotic, rifamycin antibiotic, phenicol antibiotic, triclosan	antibiotic target alteration, antibiotic efflux	91.0	100.0
INSAq77p_34	Strict	*Klebsiella pneumoniae* KpnF	protein homolog	major facilitator superfamily (MFS) antibiotic efflux pump	macrolide antibiotic, aminoglycoside antibiotic, cephalosporin, tetracycline antibiotic, peptide antibiotic, rifamycin antibiotic	antibiotic efflux	89.0	100.0
INSAq77p_3	Strict	rsmA	protein homolog	resistance-nodulation-cell division (RND) antibiotic efflux pump	fluoroquinolone antibiotic, diaminopyrimidine antibiotic, phenicol antibiotic	antibiotic efflux	85.3	100.0
INSAq77p_13	Strict	*Escherichia coli* ampH beta-lactamase	protein homolog	*ampC*-type beta-lactamase	cephalosporin, penam	antibiotic inactivation	85.2	100.8
INSAq77p_34	Strict	*Klebsiella pneumoniae* KpnE	protein homolog	major facilitator superfamily (MFS) antibiotic efflux pump	macrolide antibiotic, aminoglycoside antibiotic, cephalosporin, tetracycline antibiotic, peptide antibiotic, rifamycin antibiotic	antibiotic efflux	82.0	83.3
INSAq77p_11	Strict	adeF	protein homolog	resistance-nodulation-cell division (RND) antibiotic efflux pump	fluoroquinolone antibiotic, tetracycline antibiotic	antibiotic efflux	60.9	99.2
INSAq77p_1	Strict	*Haemophilus influenzae* PBP3 mutant (D350N, S357N)	protein variant	Penicillin-binding protein mutations conferring resistance to beta-lactam antibiotics	cephalosporin, cephamycin, penam	antibiotic target alteration	53.1	96.4
INSAq77p_9	Strict	adeF	protein homolog	resistance-nodulation-cell division (RND) antibiotic efflux pump	fluoroquinolone antibiotic, tetracycline antibiotic	antibiotic efflux	41.2	97.9

**Table 2:** Comparison of the INSAq77 *mcr-9*-containing contigs with the top nine IncHI2 *mcr-9*-harboring plasmids showing the highest identities (>99.0%, E-value 0.0, query coverage >94.0%).

Plasmid (bp)	Strain	Isolation Source/Country/Year	Identity (%)	Query Cover (%)	pMLST ^b^	Acquired Antibiotic and Desinfectant Resistance Genes ^c^	GenBank Acc. No.
**INSAq77 IncHI2** (30,314) ^a^	*E. ludwigii* INSAq77	Seabream (*Sparus aurata*)/Portugal/2018	-	-	DLST1	***mcr-9.1***	JABRPH000000000
**pSPRC-Echo1** (339,920)	*E. hormaechei* C15117	Burns unit/Australia/2007	99.99%	99.0%	DLST1	*aac(6’)-IIc*, *aph(3’’)-Ib-type*, *aph(6)-Id*, *blaSHV-12*, *bla*_TEM-1B_, *catA2-type*, *dfrA19*, ***mcr-9***, *qacE*, *qnrA1-type*, *sul1*, *sul2*, *tet(D)*	NZ_CP032842
**p525011-HI2** (354,045)	*C. freundii* 525011	unknown/China/2017	100.00%	97.0%	untyped, Nearest STs: 7,1,4,15	*aac(3)-IId-type*, *aac(6’)-aph(2’’)*, *aadA5*, *armA*, *bla*_TEM-1B_, *catA2-type*, *dfrA1-type*, ***mcr-9***, *mph(E)*, *msr(E)*, *qacE*, *qnrA1-type*, *sul1*, *sul2*	NZ_MF344582
**pOSUKPC4** (351,806)	*E. hormaechei* OSUKPC4_L	Animal/USA/2016	100.00%	98.0%	DLST1	*aadA1*, *aph(3’’)-Ib-type*, *aph(6)-Id*, *bla*_KPC-4_, *bla*_OXA-129_, *dfrA21*, ***mcr-9***, *qacE*, *sul1*, *tet(B)*	NZ_CP024910
**pOSUEC_D** (354,256)	*E. hormaechei* OSUVMCKPC4-2	Animal/USA/2016	100.00%	98.0%	DLST1	*aadA1*, *aph(3’’)-Ib-type*, *aph(6)-Id*, *bla*_KPC-4_, *bla*_OXA-129_, *dfrA21*, ***mcr-9***, *qacE*, *sul1*, *tet(B)*	NZ_CP029248
**pK29** (269,674)	*K. pneumoniae* NK29	Human/Taiwan/2001	100.00%	98.0%	DLST1	*aadA2*, *bla*_CMY-8_, *bla*_CTX-M-62-type_, *catB2*, ***mcr-9***, *qacE*, *sul1*	NC_010870
**pE1_001** (357,530)	*L. adecarboxylata* E1	Burns Unit Shower/Australia/2012	100.00%	98.0%	DLST1	*formA-type*, ***mcr-9***	NZ_CP042506
**pE11_001** (339,433)	*C. freundii* E11	Burns Unit Shower/Australia/2012	100.00%	98.0%	DLST1	*formA-type*, ***mcr-9***	NZ_CP042525
**pE61_001** (357,530)	*L. adecarboxylata* E61	Burns Unit Shower/Australia/2014	100.00%	98.0%	DLST1	*formA-type*, ***mcr-9***	NZ_CP042494
**p565_1** (263,189)	*C. freundii* 565	Human Stool/Spain/2014	99,68%	95,0%	DLST1	*aac(6’)-Ib-cr*, *aadA1*, *aadA2b-type*, *bla*_CTX-M-9_, *bla*_SHV-12_, *bla*_VIM-1_, *catA1-type*, *dfrA16*, ***mcr-9-type***, *qacE*, *qnrA1-type*, *sul1*	NZ_CP038657

^a^ Length of the *mcr-9*-containing contig. ^b^ Plasmid pMLST-2.0 Server ^c^ ResFinder-4.1 (Selected %ID threshold: 90%; selected minimum length: 60%).

## Data Availability

The authors confirm all supporting data, code and protocols have been provided within the article or through supplementary data files. The *E. ludwigii* whole genome shotgun (WGS) project has the project accession JABRPH000000000. The new *bla*_ACT-type_ nucleotide sequence was submitted to the GenBank Database as *bla*_ACT-88_ with accession number MW887657, after request of the new allele number to NCBI (https://www.ncbi.nlm.nih.gov/pathogens/submit-beta-lactamase/; accessed on 4 January 2022).

## Supplementary Materials

The following supporting information can be downloaded at: https://www.mdpi.com/article/10.3390/antibiotics11091232/s1, Figure S1: Presence of prophages in INSAq77 genome identified by PHASTER. A total of eleven prophage regions were identified, of which two regions were intact, eight regions were incomplete and one region was questionable. (a) Table with prophage characteristics; (b) Location of predicted prophages within INSAq77 contigs. Region types were marked with colors: intact (green), questionable (blue) and incomplete (red).; Table S1: Loose best-hit results (≥65% of identity), by predicted gene, obtained using the Resistance Gene Identifier (RGI).; Table S2: Results obtained from prediction of a bacteria’s pathogenicity towards human hosts using PathogenFinder (https://cge.cbs.dtu.dk/services/PathogenFinder/, accessed on 4 January 2022). Results highlighted in green are those not matching protein pathogenic families.; Table S3: Demographic and genomic characteristics of the *E. ludwigii* isolates used for the phylogenomic analysis.

## References

[1] Kempf I., Jouy E., Chauvin C. (2016). Colistin use and colistin resistance in bacteria from animals. Int. J. Antimicrob. Agents.

[2] El-Sayed Ahmed M.A.E.G., Zhong L.L., Shen C., Yang Y., Doi Y., Tian G.B. (2020). Colistin and its role in the Era of antibiotic resistance: An extended review (2000–2019). Emerg. Microbes Infect..

[3] Poirel L., Jayol A., Nordmann P. (2017). Polymyxins: Antibacterial Activity, Susceptibility Testing, and Resistance Mechanisms Encoded by Plasmids or Chromosomes. Clin. Microbiol. Rev..

[4] Andrade F.F., Silva D., Rodrigues A., Pina-Vaz C. (2020). Colistin update on its mechanism of action and resistance, present and future challenges. Microorganisms.

[5] Sun J., Zhang H., Liu Y.H., Feng Y. (2018). Towards Understanding MCR-like Colistin Resistance. Trends Microbiol..

[6] Zhang S., Abbas M., Rehman M.U., Wang M., Jia R., Chen S., Liu M., Zhu D., Zhao X., Gao Q. (2021). Updates on the global dissemination of colistin-resistant *Escherichia coli*: An emerging threat to public health. Sci. Total Environ..

[7] Ling Z., Yin W., Shen Z., Wang Y., Shen J., Walsh T.R. (2020). Epidemiology of mobile colistin resistance genes *mcr-1* to *mcr-9*. J. Antimicrob. Chemother..

[8] Carroll L.M., Gaballa A., Guldimann C., Sullivan G., Henderson L.O., Wiedmanna M. (2019). Identification of Novel Mobilized Colistin Resistance Gene *mcr-9* in a Multidrug-Resistant, Colistin-Susceptible *Salmonella enterica* Serotype Typhimurium Isolate. MBio.

[9] Wang C., Feng Y., Liu L., Wei L., Kang M., Zong Z. (2020). Identification of novel mobile colistin resistance gene *mcr-10*. Emerg. Microbes. Infect..

[10] Shen Y., Zhang R., Schwarz S., Wu C., Shen J., Walsh T.R., Wang Y. (2020). Farm animals and aquaculture: Significant reservoirs of mobile colistin resistance genes. Environ. Microbiol..

[11] Cabello F.C., Godfrey H.P. (2018). Aquaculture, exaptation, and the origin of mcr-positive colistin resistance. Antimicrob. Agents Chemother..

[12] Cabello F.C., Tomova A., Ivanova L., Godfrey H.P. (2017). Aquaculture and *mcr* colistin resistance determinants. MBio.

[13] Lei T., Zhang J., Jiang F., He M., Zeng H., Chen M., Wu S., Wang J., Ding Y., Wu Q. (2019). First detection of the plasmid-mediated colistin resistance gene *mcr-1* in virulent *Vibrio parahaemolyticus*. Int. J. Food Microbiol..

[14] Lv L., Cao Y., Yu P., Huang R., Wang J., Wen Q., Zhi C., Zhang Q., Liu J.-H. (2018). Detection of *mcr-1* Gene among *Escherichia coli* Isolates from Farmed Fish and Characterization of *mcr-1*-Bearing IncP Plasmids. Antimicrob. Agents Chemother..

[15] Hoa T.T.T., Nakayama T., Huyen H.M., Harada K., Hinenoya A., Phuong N.T., Yamamoto Y. (2020). Extended-spectrum beta-lactamase-producing *Escherichia coli* harbouring *sul* and *mcr-1* genes isolates from fish gut contents in the Mekong Delta, Vietnam. Lett. Appl. Microbiol..

[16] Yamaguchi T., Kawahara R., Harada K., Teruya S., Nakayama T., Motooka D., Nakamura S., Do Nguyen P., Kumeda Y., Van Dang C. (2018). The presence of colistin resistance gene *mcr-1* and *-3* in ESBL producing *Escherichia coli* isolated from food in Ho Chi Minh City, Vietnam. FEMS Microbiol. Lett..

[17] Lozano-Leon A., Garcia-Omil C., Dalama J., Rodriguez-Souto R., Martinez-Urtaza J., Gonzalez-Escalona N. (2019). Detection of colistin resistance *mcr-1* gene in *Salmonella enterica* serovar Rissen isolated from mussels, Spain, 2012- to 2016. Euro Surveill..

[18] Hassan J., Eddine R.Z., Mann D., Li S., Deng X., Saoud I.P., Kassem I.I. (2020). The mobile colistin resistance gene, *mcr-1.1*, is carried on Incx4 plasmids in multidrug resistant *E. coli* isolated from rainbow trout aquaculture. Microorganisms.

[19] Kalová A., Gelbíčová T., Overballe-Petersen S., Litrup E., Karpíšková R. (2021). Characterisation of colistin-resistant *Enterobacterales* and *Acinetobacter* strains carrying *mcr* genes from asian aquaculture products. Antibiotics.

[20] Xu C., Lv Z., Shen Y., Liu D., Fu Y., Zhou L., Liu W., Chen K., Ye H., Xia X. (2020). Metagenomic insights into differences in environmental resistome profiles between integrated and monoculture aquaculture farms in China. Environ. Int..

[21] Li Y., Dai X., Zeng J., Gao Y., Zhang Z., Zhang L. (2020). Characterization of the global distribution and diversified plasmid reservoirs of the colistin resistance gene *mcr-9*. Sci. Rep..

[22] Wu W., Feng Y., Zong Z. (2020). Precise Species Identification for *Enterobacter*: A Genome Sequence-Based Study with Reporting of Two Novel Species, *Enterobacter quasiroggenkampii* sp. nov. and *Enterobacter quasimori* sp. nov. mSystems.

[23] Mateos M., Hernández-García M., Del Campo R., Martínez-García L., Gijón D., Morosini M.I., Ruiz-Garbajosa P., Cantón R. (2021). Emergence and Persistence over Time of Carbapenemase-Producing *Enterobacter* Isolates in a Spanish University Hospital in Madrid, Spain (2005-2018). Microb. Drug Resist..

[24] Mezzatesta M.L., Gona F., Stefani S. (2012). *Enterobacter cloacae* complex: Clinical impact and emerging antibiotic resistance. Future Microbiol..

[25] Hoffmann H., Stindl S., Stumpf A., Mehlen A., Monget D., Heesemann J., Schleifer K.H., Roggenkamp A. (2005). Description of *Enterobacter ludwigii* sp. nov., a novel *Enterobacter* species of clinical relevance. Syst. Appl. Microbiol..

[26] Davin-Regli A., Lavigne J.P., Pagès J.M. (2019). *Enterobacter* spp.: Update on taxonomy, clinical aspects, and emerging antimicrobial resistance. Clin. Microbiol. Rev..

[27] Ali A., Sultan I., Mondal A.H., Siddiqui M.T., Gogry F.A., Haq Q.M.R. (2021). Lentic and effluent water of Delhi-NCR: A reservoir of multidrug-resistant bacteria harbouring *bla*CTX-M, *bla*TEM and *bla*SHV type ESBL genes. J. Water Health.

[28] Preena P.G., Dharmaratnam A., Raj N.S., Raja S.A., Nair R.R., Swaminathan T.R. (2021). Antibiotic-resistant *Enterobacteriaceae* from diseased freshwater goldfish. Arch. Microbiol..

[29] Lee K.E., Adhikari A., Kang S.M., You Y.H., Joo G.J., Kim J.H., Kim S.J., Lee I.J. (2019). Isolation and characterization of the high silicate and phosphate solubilizing novel strain *Enterobacter ludwigii* GAK2 that promotes growth in rice plants. Agronomy.

[30] Porto de Souza Vandenberghe L., Marcela Blandon Garcia L., Rodrigues C., Cândido Camara M., Vinícius de Melo Pereira G., de Oliveira J., Ricardo Soccol C. (2017). Potential applications of plant probiotic microorganisms in agriculture and forestry. AIMS Microbiol..

[31] Börjesson S., Greko C., Myrenås M., Landén A., Nilsson O., Pedersen K. (2020). A link between the newly described colistin resistance gene *mcr-9* and clinical *Enterobacteriaceae* isolates carrying *bla*SHV-12 from horses in Sweden. J. Glob. Antimicrob. Resist..

[32] Tyson G.H., Li C., Hsu C.H., Ayers S., Borenstein S., Mukherjee S., Tran T.T., McDermot P.F., Zhao S. (2020). The *mcr-9* gene of *Salmonella* and *Escherichia coli* is not associated with colistin resistance in the United States. Antimicrob. Agents Chemother..

[33] Zhang H., Zhao D., Quan J., Hua X., Yu Y. (2019). *mcr-1* facilitated selection of high-level colistin-resistant mutants in *Escherichia coli*. Clin. Microbiol. Infect..

[34] Zhu X.Q., Liu Y.Y., Wu R., Xun H., Sun J., Li J., Feng Y., Liu J.H. (2021). Impact of *mcr-1* on the Development of High Level Colistin Resistance in *Klebsiella pneumoniae* and *Escherichia coli*. Front. Microbiol..

[35] Ferreira I., Beisken S., Lueftinger L., Weinmaier T., Klein M., Bacher J., Patel R., von Haeseler A., Posch A.E. (2020). Species identification and antibiotic resistance prediction by analysis of whole-genome sequence data by use of ARESdb: An analysis of isolates from the unyvero lower respiratory tract infection trial. J. Clin. Microbiol..

[36] Alcock B.P., Raphenya A.R., Lau T.T.Y., Tsang K.K., Bouchard M., Edalatmand A., Huynh W., Nguyen A.L.V., Cheng A.A., Liu S. (2020). CARD 2020: Antibiotic resistome surveillance with the comprehensive antibiotic resistance database. Nucleic Acids Res..

[37] Schwarz S., Kehrenberg C., Doublet B., Cloeckaert A. (2004). Molecular basis of bacterial resistance to chloramphenicol and florfenicol. FEMS Microbiol. Rev..

[38] Li J., Zhang H., Ning J., Sajid A., Cheng G., Yuan Z., Hao H. (2019). The nature and epidemiology of OqxAB, a multidrug efflux pump. Antimicrob. Resist. Infect. Control.

[39] Fernández L., Hancock R.E.W. (2012). Adaptive and mutational resistance: Role of porins and efflux pumps in drug resistance. Clin. Microbiol. Rev..

[40] Kümmerle N., Feucht H.H., Kaulfers P.M. (1996). Plasmid-mediated formaldehyde resistance in *Escherichia coli*: Characterization of resistance gene. Antimicrob. Agents Chemother..

[41] Cloete T.E. (2003). Resistance mechanisms of bacteria to antimicrobial compounds. Int. Biodeterior. Biodegrad..

[42] Taylor D.E. (1999). Bacterial tellurite resistance. Trends Microbiol..

[43] Partridge S.R., Kwong S.M., Firth N., Jensen S.O. (2018). Mobile genetic elements associated with antimicrobial resistance. Clin. Microbiol. Rev..

[44] Mikalová L., Bosák J., Hříbková H., Dědičová D., Benada O., Šmarda J., Šmajs D. (2017). Novel temperate phages of *Salmonella enterica* subsp. *salamae* and subsp. *diarizonae* and their activity against pathogenic *S. enterica* subsp. *enterica* isolates. PLoS ONE.

[45] Jakhetia R., Talukder K.A., Verma N.K. (2013). Isolation, characterization and comparative genomics of bacteriophage SfIV: A novel serotype converting phage from *Shigella flexneri*. BMC Genom..

[46] Pan Y., Fang Y., Feng Y., Lyu N., Chen L., Li J., Xu X., Zhu B., Hu Y. (2021). Discovery of *mcr-3.1* gene carried by a prophage located in a conjugative IncA/C2 plasmid from a *Salmonella* Choleraesuis clinical isolate. J. Infect..

[47] Zhang C., Feng Y., Liu F., Jiang H., Qu Z., Lei M., Wang J., Zhang B., Hu Y., Ding J. (2017). A Phage-Like IncY Plasmid Carrying the *mcr-1* Gene in *Escherichia coli* from a Pig Farm in China. Antimicrob. Agents Chemother..

[48] Wang X., Wood T.K. (2016). Cryptic prophages as targets for drug development. Drug Resist. Updat..

[49] Arndt D., Grant J.R., Marcu A., Sajed T., Pon A., Liang Y., Wishart D.S. (2016). PHASTER: A better, faster version of the PHAST phage search tool. Nucleic Acids Res..

[50] Kizny Gordon A., Phan H.T.T., Lipworth S.I., Cheong E., Gottlieb T., George S., Peto T.E.A., Mathers A.J., Walker A.S., Crook D.W. (2020). Genomic dynamics of species and mobile genetic elements in a prolonged *bla*IMP-4-associated carbapenemase outbreak in an Australian hospital. J. Antimicrob. Chemother..

[51] Kieffer N., Royer G., Decousser J.-W., Bourrel A.-S., Palmieri M., Ortiz De La Rosa J.-M., Jacquier H., Denamur E., Nordmann P., Poirel L. (2019). *mcr-9*, an Inducible Gene Encoding an Acquired Phosphoethanolamine Transferase in *Escherichia coli*, and Its Origin. Antimicrob. Agents Chemother..

[52] Bitar I., Papagiannitsis C.C., Kraftova L., Chudejova K., Mattioni Marchetti V., Hrabak J. (2020). Detection of Five *mcr-9* -Carrying *Enterobacterales* Isolates in Four Czech Hospitals. mSphere.

[53] Khedher M.B., Baron S.A., Riziki T., Ruimy R., Raoult D., Diene S.M., Rolain J.M. (2020). Massive analysis of 64,628 bacterial genomes to decipher water reservoir and origin of mobile colistin resistance genes: Is there another role for these enzymes?. Sci. Rep..

[54] Xiaomin S., Yiming L., Yuying Y., Zhangqi S., Yongning W., Shaolin W. (2020). Global impact of *mcr-1*-positive *Enterobacteriaceae* bacteria on “one health”. Crit. Rev. Microbiol..

[55] Salgueiro V., Manageiro V., Bandarra N.M., Reis L., Caniça M. (2020). Bacterial Diversity and Antibiotic Susceptibility of *Sparus aurata* from Aquaculture. Microorganisms.

[56] Braak K., Schrijver R., Bergevoet R., European Commission Directorate-General for Health and Food Safety (2017). Welfare of Farmed Fish: Common Practices during Transport and at Slaughter: Executive Summary.

[57] Ciufo S., Kannan S., Sharma S., Badretdin A., Clark K., Turner S., Brover S., Schoch C.L., Kimchi A., DiCuccio M. (2018). Using average nucleotide identity to improve taxonomic assignments in prokaryotic genomes at the NCBI. Int. J. Syst. Evol. Microbiol..

[58] Camacho C., Coulouris G., Avagyan V., Ma N., Papadopoulos J., Bealer K., Madden T.L. (2009). BLAST+: Architecture and applications. BMC Bioinform..

[59] Varani A.M., Siguier P., Gourbeyre E., Charneau V., Chandler M. (2011). ISsaga is an ensemble of web-based methods for high throughput identification and semi-automatic annotation of insertion sequences in prokaryotic genomes. Genome Biol..

[60] Sullivan M.J., Petty N.K., Beatson S.A. (2011). Easyfig: A genome comparison visualizer. Bioinformatics.

[61] Cherak Z., Loucif L., Moussi A., Rolain J.M. (2021). Epidemiology of mobile colistin resistance (*mcr*) genes in aquatic environments. J. Glob. Antimicrob. Resist..

