# Supplementary material for: Genomic Analysis of a mcr-9.1-Harbouring IncHI2-ST1 Plasmid from Enterobacter ludwigii Isolated in Fish Farming
Source: Antibiotics (Basel). 2022 Sep 10;11(9):1232. doi: 10.3390/antibiotics11091232 (PMC9495039; doi:10.3390/antibiotics11091232)
Supplement: Supplementary file 1 [file antibiotics-11-01232-s001.zip › Table S1.pdf]

Table S1. Loose best-hit results (≥65% of identity), by predicted gene, obtained using the Resistance Gene Identifier (RGI).

Contig	RGI Criteria	ARO Term	Detection Criteria Model	AMR Gene Family	Drug Class	Resistance Mechanism	% Identity of Matching Region	% Length of Reference Sequence
INSAq77p_72	Loose	ramA	protein homolog	resistance-nodulation-cell division (RND) antibiotic efflux pump; General Bacterial Porin with reduced permeability to beta-lactams	fluoroquinolone; monobactam; carbapenem; cephalosporin; glycylcycline; cephamycin; penam; tetracycline; rifamycin; phenicol; triclosan; penem	antibiotic efflux; reduced permeability to antibiotic	97.4	91.1
INSAq77p_3	Loose	acrA	protein homolog	resistance-nodulation-cell division (RND) antibiotic efflux pump	fluoroquinolone; cephalosporin; glycylcycline; penam; tetracycline; rifamycin; phenicol; triclosan	antibiotic efflux	95.5	100.0
INSAq77p_87	Loose	cpxA	protein homolog	resistance-nodulation-cell division (RND) antibiotic efflux pump	aminoglycoside; aminocoumarin	antibiotic efflux	94.8	100.0
INSAq77p_44	Loose	bacA	protein homolog	undecaprenyl pyrophosphate related proteins	peptide	antibiotic target alteration	94.1	100.0
INSAq77p_42	Loose	soxR	protein overexpression	ATP-binding cassette (ABC) antibiotic efflux pump; major facilitator superfamily (MFS) antibiotic efflux pump; resistance-nodulation-cell division (RND) antibiotic efflux pump	fluoroquinolone; cephalosporin; glycylcycline; penam; tetracycline; rifamycin; phenicol; triclosan	antibiotic target alteration; antibiotic efflux	93.4	98.7
INSAq77p_2	Loose	acrB	protein homolog	resistance-nodulation-cell division (RND) antibiotic efflux pump	fluoroquinolone; cephalosporin; glycylcycline; penam; tetracycline; rifamycin; phenicol; triclosan	antibiotic efflux	92.9	99.9
INSAq77p_3	Loose	mdtC	protein homolog	resistance-nodulation-cell division (RND) antibiotic efflux pump	aminocoumarin	antibiotic efflux	91.4	100.0
INSAq77p_18	Loose	YojI	protein homolog	ATP-binding cassette (ABC) antibiotic efflux pump	peptide	antibiotic efflux	90.1	100.0
INSAq77p_2	Loose	mdtB	protein homolog	resistance-nodulation-cell division (RND) antibiotic efflux pump	aminocoumarin	antibiotic efflux	90.0	100.0
INSAq77p_41	Loose	soxS	protein overexpression	ATP-binding cassette (ABC) antibiotic efflux pump; major facilitator superfamily (MFS) antibiotic efflux pump; resistance-nodulation-cell division (RND) antibiotic efflux pump; General Bacterial Porin with reduced permeability to beta-lactams	fluoroquinolone; monobactam; carbapenem; cephalosporin; glycylcycline; cephamycin; penam; tetracycline; rifamycin; phenicol; triclosan; penem	antibiotic target alteration; antibiotic efflux; reduced permeability to antibiotic	89.7	100.9
INSAq77p_6	Loose	KpnG	protein homolog	major facilitator superfamily (MFS) antibiotic efflux pump	macrolide; fluoroquinolone; aminoglycoside; carbapenem; cephalosporin; penam; peptide; penem	antibiotic efflux	87.4	100.0
INSAq77p_3	Loose	mdtG	protein homolog	major facilitator superfamily (MFS) antibiotic efflux pump	fosfomycin	antibiotic efflux	86.7	100.3
INSAq77p_83	Loose	mdfA	protein homolog	major facilitator superfamily (MFS) antibiotic efflux pump	tetracycline; benzalkonium chloride; rhodamine	antibiotic efflux	86.2	114.4
INSAq77p_5	Loose	baeS	protein homolog	resistance-nodulation-cell division (RND) antibiotic efflux pump	aminoglycoside; aminocoumarin	antibiotic efflux	86.1	100.0
INSAq77p_20	Loose	OmpA	protein homolog	General Bacterial Porin with reduced permeability to beta-lactams	monobactam; carbapenem; cephalosporin; cephamycin; penam; penem	reduced permeability to antibiotic	86.0	93.9

Contig	RGI Criteria	ARO Term	Detection Criteria Model	AMR Gene Family	Drug Class	Resistance Mechanism	% Identity of Matching Region	% Length of Reference Sequence
INSAq77p_6	Loose	MdtK	protein homolog	multidrug and toxic compound extrusion (MATE) transporter	fluoroquinolone	antibiotic efflux	85.8	101.3
INSAq77p_13	Loose	mdtH	protein homolog	major facilitator superfamily (MFS) antibiotic efflux pump	fluoroquinolone	antibiotic efflux	85.1	100.0
INSAq77p_32	Loose	TolC	protein homolog	ATP-binding cassette (ABC) antibiotic efflux pump; major facilitator superfamily (MFS) antibiotic efflux pump; resistance-nodulation-cell division (RND) antibiotic efflux pump	macrolide; fluoroquinolone; aminoglycoside; carbapenem; cephalosporin; glycylcycline; cephamycin; penam; tetracycline; peptide; aminocoumarin; rifamycin; phenicol; triclosan; penem	antibiotic efflux	84.9	99.4
INSAq77p_188	Loose	PmrF	protein homolog	pmr phosphoethanolamine transferase	peptide	antibiotic target alteration	84.8	101.6
INSAq77p_118	Loose	kdpE	protein homolog	kdpDE	aminoglycoside	antibiotic efflux	84.4	100.0
INSAq77p_46	Loose	AcrF	protein homolog	resistance-nodulation-cell division (RND) antibiotic efflux pump	fluoroquinolone; cephalosporin; cephamycin; penam	antibiotic efflux	84.0	100.3
INSAq77p_18	Loose	ugd	protein homolog	pmr phosphoethanolamine transferase	peptide	antibiotic target alteration	83.5	100.0
INSAq77p_1	Loose	mdtA	protein homolog	resistance-nodulation-cell division (RND) antibiotic efflux pump	aminocoumarin	antibiotic efflux	81.2	96.9
INSAq77p_21	Loose	OmpK37	protein homolog	General Bacterial Porin with reduced permeability to beta-lactams	monobactam; carbapenem; cephalosporin; cephamycin; penam; penem	reduced permeability to antibiotic	80.6	98.7
INSAq77p_4	Loose	acrR	protein overexpression	resistance-nodulation-cell division (RND) antibiotic efflux pump	fluoroquinolone; cephalosporin; glycylcycline; penam; tetracycline; rifamycin; phenicol; triclosan	antibiotic target alteration; antibiotic efflux	79.8	100.9
INSAq77p_61	Loose	LptD	protein homolog	ATP-binding cassette (ABC) antibiotic efflux pump	carbapenem; peptide; aminocoumarin; rifamycin	antibiotic efflux	79.6	100.1
INSAq77p_45	Loose	AcrE	protein homolog	resistance-nodulation-cell division (RND) antibiotic efflux pump	fluoroquinolone; cephalosporin; cephamycin; penam	antibiotic efflux	74.7	98.4
INSAq77p_17	Loose	rosB	protein homolog	major facilitator superfamily (MFS) antibiotic efflux pump	peptide	antibiotic efflux	74.0	99.1
INSAq77p_17	Loose	sdiA	protein homolog	resistance-nodulation-cell division (RND) antibiotic efflux pump	fluoroquinolone; cephalosporin; glycylcycline; penam; tetracycline; rifamycin; phenicol; triclosan	antibiotic efflux	73.3	100.0
INSAq77p_153	Loose	eptB	protein homolog	pmr phosphoethanolamine transferase	peptide	antibiotic target alteration	73.2	98.3
INSAq77p_18	Loose	rosA	protein homolog	major facilitator superfamily (MFS) antibiotic efflux pump	peptide	antibiotic efflux	71.8	98.1
INSAq77p_185	Loose	ArnT	protein homolog	pmr phosphoethanolamine transferase	peptide	antibiotic target alteration	70.2	100.0
INSAq77p_187	Loose	arnA	protein homolog	pmr phosphoethanolamine transferase	peptide	antibiotic target alteration	69.7	99.7

Contig	RGI Criteria	ARO Term	Detection Criteria Model	AMR Gene Family	Drug Class	Resistance Mechanism	% Identity of Matching Region	% Length of Reference Sequence
INSAq77p_1	Loose	OmpK37	protein homolog	General Bacterial Porin with reduced permeability to beta-lactams	monobactam; carbapenem; cephalosporin; cephamycin; penam; penem	reduced permeability to antibiotic	68.1	18.7
INSAq77p_19	Loose	catA4	protein homolog	chloramphenicol acetyltransferase (CAT)	phenicol	antibiotic inactivation	65.9	106.9
INSAq77p_22	Loose	OmpK37	protein homolog	General Bacterial Porin with reduced permeability to beta-lactams	monobactam; carbapenem; cephalosporin; cephamycin; penam; penem	reduced permeability to antibiotic	65.7	106.2
