# Supplementary material for: Genomic Analysis of a mcr-9.1-Harbouring IncHI2-ST1 Plasmid from Enterobacter ludwigii Isolated in Fish Farming
Source: Antibiotics (Basel). 2022 Sep 10;11(9):1232. doi: 10.3390/antibiotics11091232 (PMC9495039; doi:10.3390/antibiotics11091232)
Supplement: Supplementary file 1 [file antibiotics-11-01232-s001.zip › Table S2.pdf]

Table S2. Results obtained from prediction of a bacteria's pathogenicity towards human hosts using PathogenFinder (https://cge.cbs.dtu.dk/services/PathogenFinder/). Results highlighted in green are those not matching protein pathogenic families.

INSAq77 Contig	NCBI Accession number	Description	Protein Function	NCBI Protein ID	Identity (%)
INSAq771p_94_1	unknown	*Salmonella enterica* subsp. *enterica* serovar Typhi str. CT18	unknown	unknown	100.0
INSAq771p_20_58	CP000880	*Salmonella enterica* subsp. arizonae serovar 62:z4,z23:--	hypothetical protein	ABX24195	97.07
INSAq771p_43_25	CU928158	*Escherichia fergusonii* ATCC 35469 chromosome	inorganic polyphosphate/ATP-NAD kinase	CAQ88017	97.6
INSAq771p_1_175	AP006725	*Klebsiella pneumoniae* NTUH-K2044 DNA	4-hydroxyphenylacetate 3-hydroxylase	BAH61578	98.08
INSAq771p_3_58	CP000964	*Klebsiella pneumoniae* 342	4-hydroxybenzoate decarboxylase, subunit C	ACI07194	96.0
INSAq771p_10_38	CP000964	*Klebsiella pneumoniae* 342	ascorbate-specific permease IIC component	ACI07087	96.78
INSAq771p_83_4	CP000948	*Escherichia coli* str. K12 substr. DH10B	CP4-6 prophage; predicted sugar transporter	ACB01437	96.73
INSAq771p_17_52	CP000822	*Citrobacter koseri* ATCC BAA-895	hypothetical protein	ABV14881	97.03
INSAq771p_70_19	CU928163	*Escherichia coli* UMN026 chromosome	putative pyridine nucleotide-disulfide oxidoreductase	CAR11557	99.55
INSAq771p_46_7	DQ517526	*Escherichia coli* APEC O1 plasmid pAPEC-O1-R	putative DNA methyltransferase	ABF67893	97.84
INSAq771p_4_46	CP000822	*Citrobacter koseri* ATCC BAA-895	hypothetical protein	ABV15790	98.73
INSAq771p_91_11	CP000650	*Klebsiella pneumoniae* subsp. pneumoniae MGH 78578 plasmid pKPN5	plasmid partition protein A	ABR80603	99.5
INSAq771p_10_82	CP000822	*Citrobacter koseri* ATCC BAA-895	hypothetical protein	ABV14677	95.78
INSAq771p_5_104	CP001138	*Salmonella enterica* subsp. *enterica* serovar Agona str. SL483	secretion protein HlyD family protein	ACH51271	95.77
INSAq771p_46_27	DQ517526	*Escherichia coli* APEC O1 plasmid pAPEC-O1-R	RepH12	ABF67907	99.45
INSAq771p_62_1	AP010960	*Escherichia coli* O111:H- str. 11128 DNA	hypothetical protein	BAI35195	98.86
INSAq771p_8_15	CP000648	*Klebsiella pneumoniae* subsp. pneumoniae MGH 78578 plasmid pKPN3	Hypothetical protein	ABR80369	97.75
INSAq771p_25_17	CP001063	*Shigella boydii* CDC 3083-94	DNA replication and repair protein RecF	ACD08678	96.36
INSAq771p_2_73	CP000468	*Escherichia coli* APEC O1	UDP-galactose-4-epimerase	ABJ00142	96.45
INSAq771p_107_4	AE014073	*Shigella flexneri* 2a str. 2457T	IS911 orfA	AAP15762	96.31
INSAq771p_61_7	CP000886	*Salmonella enterica* subsp. *enterica* serovar Paratyphi B str. SPB7	hypothetical protein	ABX66384	96.32
INSAq771p_22_45	CP001138	*Salmonella enterica* subsp. *enterica* serovar Agona str. SL483	phage integrase	ACH51627	97.36
INSAq771p_93_6	AE017042	*Yersinia pestis* biovar Microtus str. 91001	transposase for insertion sequence IS100	AAS60313	100.0
INSAq771p_7_29	CP000243	*Escherichia coli* UTI89	acetyl-coenzyme A carboxylase carboxyl transferase subunit beta	ABE08068	98.01
INSAq771p_91_12	CP000966	*Klebsiella pneumoniae* 342 plasmid pKP91	plasmid partition parB protein	ACI12297	99.38
INSAq771p_15_32	CP000243	*Escherichia coli* UTI89	30S ribosomal protein S2	ABE05693	97.93
INSAq771p_193_1	CP001383	*Shigella flexneri* 2002017	ISEhe3, transposase orfB	ADA76515	99.02

INSAq771p_91_9	CP000650	*Klebsiella pneumoniae* subsp. pneumoniae MGH 78578 plasmid pKPN5	DNA replication	ABR80601	99.31
INSAq771p_6_5	CP000266	*Shigella flexneri* 5 str. 8401	conserved hypothetical protein	ABF06037	95.75
INSAq771p_34_40	CP000822	*Citrobacter koseri* ATCC BAA-895	hypothetical protein	ABV12545	98.0
INSAq771p_1_132	CP000822	*Citrobacter koseri* ATCC BAA-895	hypothetical protein	ABV14493	97.3
INSAq771p_93_7	CP000244	*Escherichia coli* UTI89 plasmid pUTI89	putative transposase	ABE10617	100.0
INSAq771p_14_9	CP000822	*Citrobacter koseri* ATCC BAA-895	hypothetical protein	ABV15586	98.07
INSAq771p_62_3	CP001164	*Escherichia coli* O157:H7 str. EC4115	conserved hypothetical protein	ACI36787	99.22
INSAq771p_2_77	CP000880	*Salmonella enterica* subsp. arizonae serovar 62:z4,z23:--	hypothetical protein	ABX22051	96.8
INSAq771p_83_6	U00096	*Escherichia coli* str. K-12 substr. MG1655	CP4-6 prophage; predicted DNA-binding transcriptional regulator	AAC73375	95.63
INSAq771p_18_19	CP001113	*Salmonella enterica* subsp. *enterica* serovar Newport str. SL254	ribonuclease III	ACF61892	95.82
INSAq771p_13_34	CP000822	*Citrobacter koseri* ATCC BAA-895	hypothetical protein	ABV13829	98.55
INSAq771p_62_9	DQ517526	*Escherichia coli* APEC O1 plasmid pAPEC-O1-R	TerY1	ABF67743	99.06
INSAq77_MCR-9_38	CP001125	*Salmonella enterica* subsp. *enterica* serovar Schwarzengrund str. CVM19633 plasmid pCVM1963	10, complete sequence.	ACF88522	100.0
INSAq771p_113_4	AP006725	*Klebsiella pneumoniae* NTUH-K2044 DNA	fimbrial chaperone protein mrkB precursor	BAH65060	100.0
INSAq771p_10_14	CP000247	*Escherichia coli* 536	oligoribonuclease	ABG72350	96.13
INSAq771p_10_41	CP000964	*Klebsiella pneumoniae* 342	3-dehydro-L-gulonate-6-phosphate decarboxylase	ACI10848	96.76
INSAq771p_4_93	CP000783	*Enterobacter sakazakii* ATCC BAA-894	hypothetical protein	ABU79530	98.27
INSAq771p_62_10	DQ517526	*Escherichia coli* APEC O1 plasmid pAPEC-O1-R	TerX	ABF67742	100.0
INSAq771p_18_13	CP000243	*Escherichia coli* UTI89	RNA polymerase sigma E	ABE08355	98.95
INSAq771p_162_1	CP001063	*Shigella boydii* CDC 3083-94	IS1 protein InsB	ACD09517	97.1
INSAq771p_15_21	CP000822	*Citrobacter koseri* ATCC BAA-895	hypothetical protein	ABV14271	97.35
INSAq771p_3_23	CP001144	*Salmonella enterica* subsp. *enterica* serovar Dublin str. C	2021853, complete genome.	ACH75166	97.86
INSAq771p_5_36	CP001063	*Shigella boydii* CDC 3083-94	conserved hypothetical protein	ACD09359	98.33
INSAq771p_9_48	CP000034	*Shigella dysenteriae* Sd197	conserved hypothetical protein	ABB62686	96.45
INSAq771p_30_6	CP000243	*Escherichia coli* UTI89	50S ribosomal subunit protein L13	ABE09105	97.89
INSAq771p_54_12	XXX	*Salmonella enterica* subsp. *enterica* serovar Typhi str. CT18	XXX		98.16
INSAq771p_31_45	CP000783	*Enterobacter sakazakii* ATCC BAA-894	hypothetical protein	ABU79120	96.88
INSAq771p_6_20	AP006725	*Klebsiella pneumoniae* NTUH-K2044 DNA	transcriptional repressor for methionine biosynthesis	BAH61007	98.1
INSAq771p_81_8	CP000857	*Salmonella enterica* subsp. *enterica* serovar Paratyphi C strain RKS4594	co-chaperonin GroES	ACN48531	96.91
INSAq771p_62_7	DQ517526	*Escherichia coli* APEC O1 plasmid pAPEC-O1-R	TerW	ABF67744	100.0

INSAq771p_26_25	CP000948	*Escherichia coli* str. K12 substr. DH10B	CP4-6 prophage; inner membrane lipoprotein	ACB01418	100.0
INSAq771p_19_15	CP000247	*Escherichia coli* 536	thioredoxin 1	ABG71937	98.17
INSAq771p_160_3	CP000244	*Escherichia coli* UTI89 plasmid pUTI89	hypothetical protein	ABE10614	97.76
INSAq771p_119_5	CP000784	*Enterobacter sakazakii* ATCC BAA-894 plasmid pESA2	hypothetical protein	ABU79601	100.0
INSAq771p_92_4	CP000036	*Shigella boydii* Sb227	putative alpha helix protein	ABB65503	95.5
INSAq771p_29_45	AE017220	*Salmonella enterica* subsp. *enterica* serovar Choleraesuis str. SC-B67	chemotaxis regulator, transmits chemoreceptor signals to flagelllar motor components	AAX65829	95.35
INSAq771p_70_17	CP001063	*Shigella boydii* CDC 3083-94	IS1 transposase orfB	ACD09692	98.86
INSAq771p_13_64	CP000243	*Escherichia coli* UTI89	hypothetical protein YajC	ABE05930	99.09
INSAq771p_119_6	CP000784	*Enterobacter sakazakii* ATCC BAA-894 plasmid pESA2	hypothetical protein	ABU79600	100.0
INSAq77_MCR-9_32	CP000800	*Escherichia coli* E24377A	IS66 family element, orf2	ABV19481	99.13
INSAq771p_160_2	CP001063	*Shigella boydii* CDC 3083-94	IS66 family element, orf2	ACD10323	100.0
INSAq771p_26_32	CP000948	*Escherichia coli* str. K12 substr. DH10B	CP4-6 prophage; toxin of the YkfI-YafW toxin-antitoxin system	ACB01411	99.12
INSAq771p_32_43	CP000880	*Salmonella enterica* subsp. arizonae serovar 62:z4,z23:--	hypothetical protein	ABX21688	97.44
INSAq771p_10_39	AE014075	*Escherichia coli* CFT073	Unknown pentitol phosphotransferase enzyme II, B component	AAN83704	98.02
INSAq771p_8_5	CP000880	*Salmonella enterica* subsp. arizonae serovar 62:z4,z23:--	hypothetical protein	ABX21522	97.98
INSAq771p_2_165	AP006725	*Klebsiella pneumoniae* NTUH-K2044 DNA	cold shock protein	BAH62358	100.0
INSAq771p_131_1	AE014073	*Shigella flexneri* 2a str. 2457T	IS1 orfB	AAP15901	98.97
INSAq771p_91_7	CU928144	*Escherichia fergusonii* str. ATCC 35469T plasmid pEFER	hypothetical protein	CAQ86970	98.82
INSAq771p_6_137	AP006725	*Klebsiella pneumoniae* NTUH-K2044 DNA	conserved hypothetical protein	BAH60940	95.51
INSAq771p_70_18	CP000800	*Escherichia coli* E24377A	IS1, transposase orfA	ABV18481	100.0
INSAq771p_66_21	CP000799	*Escherichia coli* E24377A plasmid pETE	4, complete sequence.	ABV16448	98.9
INSAq771p_10_62	CP000026	*Salmonella enterica* subsp. *enterica* serovar Paratyphi A str. ATCC 9150	conserved hypothetical protein	AAV79961	98.53
INSAq771p_30_19	CP000034	*Shigella dysenteriae* Sd197	conserved hypothetical protein	ABB63408	97.01
INSAq771p_134_1	CP001113	*Salmonella enterica* subsp. *enterica* serovar Newport str. SL254	phage transcriptional regulator, AlpA	ACF61557	96.51
INSAq771p_26_24	CP000948	*Escherichia coli* str. K12 substr. DH10B	CP4-57 prophage; predicted inner membrane protein	ACB03777	97.44
INSAq771p_22_56	CP000026	*Salmonella enterica* subsp. *enterica* serovar Paratyphi A str. ATCC 9150	hypothetical protein	AAV78455	100.0
INSAq771p_1_137	AP006725	*Klebsiella pneumoniae* NTUH-K2044 DNA	putative amino acid/amine transport protein	BAH61631	98.11
INSAq771p_134_3	BA000007	*Escherichia coli* O157:H7 str. Sakai DNA	hypothetical protein	BAB33724	97.26
INSAq771p_73_3	CP000783	*Enterobacter sakazakii* ATCC BAA-894	hypothetical protein	ABU79426	100.0
