# Supplementary material for: Genomic Analysis of a mcr-9.1-Harbouring IncHI2-ST1 Plasmid from Enterobacter ludwigii Isolated in Fish Farming
Source: Antibiotics (Basel). 2022 Sep 10;11(9):1232. doi: 10.3390/antibiotics11091232 (PMC9495039; doi:10.3390/antibiotics11091232)
Supplement: Supplementary file 1 [file antibiotics-11-01232-s001.zip › Table S3.pdf]

Table S3. Demographic and genomic characteristics of the *E. ludwigii* isolates used for the phylogenomic analysis.

Strain	Collection Year	Location	Isolation Source	Isolation type	AMR genotypes[1]	BioSample	NCBI Accession Number	Contigs/ Scaffolds	N50 (bp)	Length (bp)
JP6	2011	China	rhizosphere soil	environmental/ other	bla_{ACT}, *catA**, *fosA*, *oqxB*	SAMN11415570	NZ_CP040256	1	4681598	4681598
JP9	2011	China	soil	environmental/ other	bla_{ACT}, *catA**, *fosA*, *oqxB*	SAMN11415807	NZ_CP040527	1	4681542	4681542
I42	2015	China	*Lycium barbarum* rhizosphere soil	environmental/ other	bla_{ACT}, *catA**, *fosA*2, *oqxB*	SAMN11415809	NZ_CP040606	1	4719369	4719369
120152	2019	China	human blood	clinical	bla_{ACT}, *catA**, *fosA*2, *oqxB*	SAMN19316194	JAHEUQ000000000	27	685439	4722419
WCHEL090041	2016	China	human	clinical	bla_{ACT}, *catA**, *fosA*, *oqxB*	SAMN09845216	RXRU00000000	38	486168	4783603
A3203	n.a.	USA	rhizosphere	environmental/ other	bla_{ACT}, *catA**, *fosA*2, *oqxB*	SAMN05589724	MSDM00000000	17	792170	4812349
WCHEL090017	2017	China	human	clinical	*aph(3'')-Ibtype*, *aph(6)-Id*, bla_{ACT}, *catA**, *fosA*2, *oqxB*	SAMN09845195	RXRA00000000	87	250730	5167660
JGM43	2015	USA	kitchen B	environmental/ other	bla_{ACT}, *catA**, *fosA*2	SAMN17146117	JAERJF000000000	97	190941	5302774
DSM16688T	n.a.	Germany	n.a.	n.a.	bla_{ACT}, *catA**, *fosA*, *oqxB*	SAMEA104113918	FYBD00000000	26	544330	4908609
LecVs2	n.a.	Italy	n.a.	n.a.	*ampC**, *oqxB*, *vat**	SAMEA3213012	CEFR00000000	197	62320	5285925
EnVs2	n.a.	Italy	n.a.	n.a.	*ampC**, *oqxB*	SAMEA3213011	CEFQ00000000	108	393305	5079786
GN02454	2005	USA	human bodily fluid	clinical	bla_{ACT}, *catA**, *fosA*, *oqxB*	SAMN03732707	LEDW00000000	22	586033	4715527
AA4	2012	USA	*Zea mays* root	environmental/ other	bla_{ACT}, *catA**, *fosA*, *oqxB*	SAMN06130962	CP018785	1	4799256	4799256
GN02730	2008	USA	human	clinical	bla_{ACT}, *catA**, *fosA*2, *oqxB*	SAMN03732724	LEER00000000	75	322082	5204500
GN02226	2003	USA	human bodily fluid	clinical	bla_{ACT}, *catA**, *fosA*, *oqxB*	SAMN03732688	LEEO00000000	36	276882	4836923
EnVs6	n.a.	Italy			ampC*, *oqxB*, vat*	SAMEA3213010	CEFO00000000	64	208468	5220112
MGYG-HGUT-02503	n.a.	Australia	human gut	clinical	bla_{ACT}, *catA**, *fosA*2, *oqxB*	SAMEA5852008	CABMNI000000000	26	332883	4697885
BIDMC121	n.a.	USA	human	clinical	bla_{ACT}, *catA**, *fosA*, *oqxB*	SAMN08148284	JACRRX000000000	30	284260	5224129
EcWSU1	2007/2008	USA	onion bulbs	environmental/ other	bla_{ACT}, *catA**, *fosA*2, *oqxB*	SAMN02604307	CP002886	2	4734438	4798091
OLC-1682	2014	Canada	n.a.	food	bla_{ACT}, *catA**, *oqxB*	SAMN03292329	JXWK00000000	1341	5354	4910606
e1617	2007	United Kingdom	human blood	clinical	bla_{ACT}, *catA**, *fosA*, *oqxB*	SAMEA2273503	FJZU00000000	37	229677	4784689
e864	2004	United Kingdom	human blood	clinical	bla_{ACT}, *catA**, *fosA*, *oqxB*	SAMEA2273255	FKHG00000000	36	234939	4786532
PDC34	n.a.	USA	n.a.	n.a.	bla_{ACT}, *catA**, *fosA*, *oqxB*	SAMN05428986	FRCI00000000	19	697266	4683223
Hanford	2008	USA	water Sample	environmental/ other	bla_{ACT}, $bla_{TEM-116}$, *catA*1, *catA**, *fosA*, *oqxB*	SAMN02212350	ATCK00000000	62	640760	4832153
MGH160	2015	USA	human	clinical	bla_{ACT}, bla_{FONA}, *catA**, *fosA*, *oqxB*	SAMN04521909	NGRN00000000	59	233137	4925300
DLL7524	2015	Australia	human feces	clinical	bla_{ACT}, *catA**, *fosA*2, *oqxB*	SAMN08374129	PQCT00000000	26	332883	4697885
P101	n.a.	USA	switchgrass	environmental/ other	bla_{ACT-12}, *catA**, *fosA*2, *oqxB*	SAMN02641647	NZ_CP006580	1	5369929	5369929
e1026	2005	United Kingdom	human blood	clinical	bla_{ACT}, *catA**, *fosA*2, *oqxB*	SAMEA2273485	FJWD00000000	27	494790	4725159

e2350	2010	United Kingdom	human blood	clinical	*bla*$_{ACT}$, *bla*$_{NMC-A}$, *catA**, *fosA*, *oqxB*	SAMEA2273452	FKCG00000000	40	491260	4963845
EN-119	n.a.	China	human	clinical	*bla*$_{ACT}$, *catA**, *fosA*, *oqxB*	SAMN05787341	NZ_CP017279	2	4857439	4952770
E8	2015	Germany	cucumber	environmental/ other	*bla*$_{ACT}$, *catA**, *fosA*, *oqxB*, $_{tet(A)-type}$	SAMN12560200	VTUA00000000	140	496749	4848102
NR1491	2013	Japan	n.a.	n.a.	*bla*$_{ACT}$-54, *bla*NMC-A, *catA**, *fosA*, *oqxB*	SAMD00184384	BKZO00000000	32	456530	4785407
ES-1	2015	USA	soil	environmental/ other	*bla*$_{ACT}$, *catA**, *fosA*, *oqxB*	SAMN11281800	SZVD00000000	95	129134	4858644
AS012248	2015	USA	human lung	clinical	*bla*$_{ACT}$, *catA**, *fosA*, *oqxB*	SAMN12250567	VLNN00000000	739	71890	5204705
AS012244	2015	USA	human lung	clinical	*bla*$_{ACT}$, *catA**, *fosA*, *oqxB*	SAMN12250563	VLNO00000000	548	209857	4972320
D42-sc-1712201	n.a.	Switzerland	n.a.	n.a.	*bla*$_{ACT}$, *catA**, *fosA2*, *oqxB*	SAMN15300311	NZ_CP056119	1	4875486	4875486
FDAARGOS 1436	2004	Germany	human mid-stream urine	clinical	*bla*$_{ACT}$, *fosA*, *oqxA*, *oqxB*	SAMN16357578	CP077223	1	4943437	4943437
K37	2020	China	human faeces	clinical	*bla*$_{ACT}$, *fosA2*, *oqxA*, *oqxB*	SAMN14389561	JAASIR000000000	17	667499	4902033
K32	2020	China	human faeces	clinical	*bla*$_{ACT}$, *fosA2*, *oqxA*, *oqxB*	SAMN14389555	JAASIL000000000	18	721734	4736013
K31_2	2020	China	human faeces	clinical	*bla*$_{ACT}$, *fosA2*, *oqxA*, *oqxB*	SAMN14389554	JAASIK000000000	15	721734	4736350
FDAARGOS_1475	2002	Greece	soil from a land farm for treatment of refinery waste sludge	environmental/ other	*bla*$_{ACT}$, *fosA2*, *oqxA*, *oqxB*	SAMN20888892	CP082860	1	4730716	4730716
FDAARGOS_1498	2002	Greece	soil from a land farm for treatment of refinery waste sludge	environmental/ other	*bla*$_{ACT}$, *catA**, *fosA2*, *oqxB*	SAMN21218854	CP083640	1	4730711	4730711
UW5	1994	Canada	soil	environmental/ other	*bla*$_{ACT}$, *catA**, *fosA*, *oqxB*	SAMN03743787	NZ_CP011798	1	4904981	4904981
2485STDY5438318	n.a.	United Kingdom	human	clinical	*bla*$_{ACT}$, *catA**, *fosA*, *oqxB*	SAMEA1964557	UNVN00000000	35	539931	4810044
2485STDY5438320	n.a.	United Kingdom	human	clinical	*bla*$_{ACT}$, *catA**, *fosA*, *oqxB*	SAMEA2053720	UNVX00000000	23	540025	4761804
EC57	2018	Japan	human blood	clinical	*bla*$_{ACT}$, *catA**, *fosA*, *oqxB*	SAMN16911616	JADRHX000000000	32	314090	4721280
MML25	2018	Japan	human blood	clinical	*bla*$_{ACT}$, *catA**, *fosA2*, *oqxB*	SAMN16911600	JADRIN000000000	22	697873	4737023
OLC-1683	2014	Canada	n.a.	food	*fosA2*, *oqxB*-type	SAMN03292330	JXWL00000000	955	8888	5136359
AOUC-8/14	2014	Italy	human rectal swab from female	clinical	*bla*$_{ACT}$, *bla*$_{NMC-A}$, *catA**, *fosA2*, *oqxB*	SAMN03861996	LGIV00000000	15	695071	4766022
GN04920	2012	USA	n.a.	clinical	*bla*$_{ACT}$, *catA**, *fosA*, *oqxB*	SAMN04572595	LVTW00000000	92	82088	4924730
NCR3	2014	Australia	*Carpobrotus rossii* rhizosphere	environmental/ other	*bla*$_{ACT}$, *catA**, *fosA2*, *oqxB*	SAMN05462048	MCGF00000000	23	771694	4779415
4928STDY7071139	2018	United Kingdom	human faecal	clinical	*bla*$_{ACT}$, *catA**, *fosA2*, *oqxB*	SAMEA104567250	CABGVX000000000	48	285987	5138127
4928STDY7071138	2018	United Kingdom	human faecal	clinical	*bla*$_{ACT}$, *catA**, *fosA2*, *oqxB*	SAMEA104567249	CABGVW000000000	47	209911	5136800
CEB04	2003	Sweden	human urinary tract catheters	clinical	*bla*$_{ACT}$, *catA**, *fosA*, *oqxB*	SAMN11357407	NZ_CP039741	1	4892375	4892475
2021EL-00127	2020	USA	human urine	clinical	*bla*$_{ACT}$, *bla*$_{NMC-A}$, *catA**, *fosA2*, *oqxB*	SAMN18511098	JAGKLL000000000	20	585339	4892341
2020EL-00108	2020	USA	human peritoneum	clinical	*bla*$_{ACT}$, *bla*$_{NMC-A}$, *catA**, *fosA2*, *oqxB*	SAMN18511080	JAGKMD000000000	38	336354	4793318
4928STDY7071136	2018	United Kingdom	human faecal	clinical	*bla*$_{ACT}$, *catA**, *fosA2*, *oqxB*	SAMEA104567247	CABGVO000000000	47	286026	5137156

4928STDY7071140	2018	United Kingdom	human faecal	clinical	*bla*$_{\text{ACT}}$, *catA**, *fosA*2, *oqxB*	SAMEA104567251	CABGVZ000000000	59	209917	5061284
I140	2017	USA	human hospital patient	clinical	*bla*$_{\text{ACT-12}}$, *catA**, *fosA*7, *fosA*, *oqxB*	SAMN15689507	JACJHJ000000000	25	542092	5039619
Res13-Abat-PEB19-P1-02-A	2017	Canada	swab	Environmental/ other	*bla*$_{\text{ACT-12}}$, *catA**, *fosA*2, *oqxB*	SAMN16304044	JADAJZ000000000	2	4656584	4661993
MGH216	n.a.	USA	human	clinical	*bla*$_{\text{ACT}}$, *catA**, *fosA*, *oqxB*	SAMN08148254	JACRRJ000000000	35	252605	5225825
608_ECLO	n.a.	USA	human	clinical	*bla*$_{\text{ACT}}$, *catA**, *fosA*, *oqxB*	SAMN03197808	JVAG00000000	222	48185	4804191
GN03638	2010	USA	human	clinical	*bla*$_{\text{ACT}}$, *catA**, *fosA*2, *oqxB*-type	SAMN04407776	LRCI00000000	167	47667	5003459
e558	2003	United Kingdom	human blood	clinical	*bla*$_{\text{ACT}}$, *catA**, *fosA*2, *oqxB*	SAMEA2273189	FKEY00000000	62	158439	5091497
48	2017	Germany	human clinical specimen	clinical	*bla*$_{\text{ACT}}$, *catA**, *fosA*2	SAMN12258083	VKFK00000000	200	203750	5134960
49	2017	Germany	human clinical specimen	clinical	*bla*$_{\text{ACT}}$, *catA**, *fosA*2	SAMN12258084	VKFJ00000000	152	185970	5122530
AS012471	2015	USA	human lung	clinical	*bla*$_{\text{ACT}}$, *catA**, *fosA*, *oqxB*	SAMN12250790	VLMA00000000	754	245975	5153193
INSAq77	2018	Portugal	*Sparus aurata*	environmental/ other	*bla*$_{\text{ACT}}$-88, *catA**, *fosA*, *mcr-9.1*, *oqxB*	SAMN15015462	JABRPH000000000	225	74544	5276953
EC56	2018	Japan	human blood	clinical	*bla*$_{\text{ACT}}$, *catA**, *fosA*2, *mcr-10.1*, *oqxB*	SAMN16911615	JADRHY000000000	80	202845	5110004
EC49	2018	Japan	human blood	clinical	*bla*$_{\text{ACT}}$, *catA**, *fosA*2, *oqxB*	SAMN16911589	JADRIY000000000	68	186338	4853579
11894	2011	China	human throat swab	clinical	*bla*$_{\text{ACT}}$, *catA**, *fosA*, *mcr-10*, *oqxB*	SAMN18435831	JAGFWU000000000	120	141921	4749540
40513	2015	France	*Corvus*	environmental/ other	*aac(6')-Ib3*, *aadA2*, *ant(2'')-Ia*, *bla*$_{\text{ACT}}$, *bla*$_{\text{CTX-M-9}}$, *bla*$_{\text{SHV-12}}$, *catA*1, *catA**, *fosA*2, *mcr-9.1*, *oqxB*, *qnrA1*, *sul1*, *tet(A)*	SAMN15925369	JAGDFR000000000	62	529698	5031188
40508	2015	France	*Pica pica*	environmental/ other	*aac(6')-Ib3*, *aadA2*, *ant(2'')-Ia*, *bla*$_{\text{ACT}}$, *bla*$_{\text{CTX-M-9}}$, *bla*$_{\text{SHV-12}}$, *catA*1, *catA**, *fosA*2, *mcr-9.1*, *oqxB*, *qnrA1*, *sul1*, *tet(A)*	SAMN15925368	JAGDFS000000000	66	423627	5031210
Y05	2020	China	human faeces	clinical	*aac(6')-Ib-cr5*, *aadA16*, *aph(3'')-Ib*, *aph(6)-Id*, *arr-3*, *bla*$_{\text{ACT}}$, *dfrA27*, *fosA*2, *mph(A)*, *oqxA*, *oqxB*, *qnrB6*, *sul1*, *tet(C)*	SAMN14389599	JAASKD000000000	49	281183	5549279
Y09	2020	China	human faeces	clinical	*aac(6')-Ib-cr5*, *aadA2*, *aph(3'')-Ib*, *aph(6)-Id*, *arr-3*, *bla*$_{\text{ACT}}$, *bla*$_{\text{OXA-1}}$, *catB3*, *dfrA12*, *dfrA14*, *fosA*3, *fosA*, *mph(A)*, *oqxA*, *oqxB*, *qnrA1*, *qnrS1*, *sul1*, *sul2*, *tet(B)*, *tet(D)*	SAMN14389603	JAASKH000000000	41	534659	5406832
AS012405	2016	Austria	human lung	clinical	*bla*$_{\text{ACT-12-type}}$, *fosA*2-type, *oqxA*-type, *oqxB*-type	SAMN12250724	VLMJ01000000	2375	182725	5650902

* (HMM): According with the NCBI Pathogen Detection (https://www.ncbi.nlm.nih.gov/pathogens), using hidden Markov models (HMMs) to identify protein families

n.a.: data not available
