# Supplementary figures and images for: Genomic Analysis of a mcr-9.1-Harbouring IncHI2-ST1 Plasmid from Enterobacter ludwigii Isolated in Fish Farming
Source: Antibiotics (Basel). 2022 Sep 10;11(9):1232. doi: 10.3390/antibiotics11091232 (PMC9495039; doi:10.3390/antibiotics11091232)

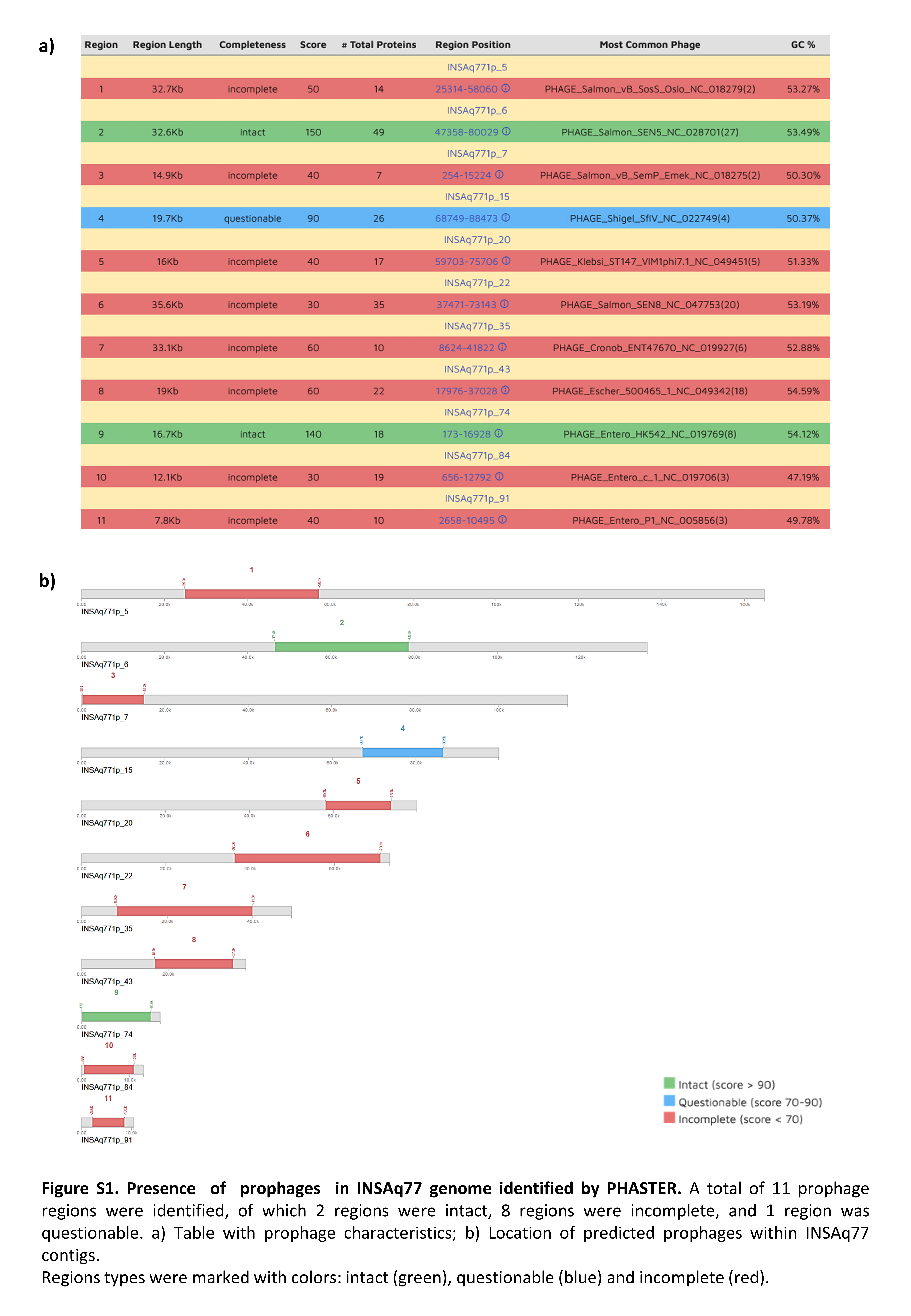

Supplement: Supplementary file 1 [file antibiotics-11-01232-s001.zip › FigS1.png]
